# N^6^-methyladenosine modification regulates cell death in cognitive impairment

**DOI:** 10.4103/NRR.NRR-D-25-00813

**Published:** 2025-09-29

**Authors:** Yiqun Li, Yuxin Zhang, Yanzhen Wang, Ke Ye, Lulu Liu, Mengjie Tian, Xinyu Han, Xinyi Chen, Tianhu Zheng, Fuyuan Li, Xu Gao, Qing Xia, Dayong Wang

**Affiliations:** 1 Department of Biochemistry and Molecular Biology, School of Basic Medical Sciences, Harbin Medical University, Harbin, Heilongjiang Province, China; 2 The First Affiliated Hospital of Harbin Medical University, Harbin, Heilongjiang Province, China; 3 Center for Endemic Disease Control, Chinese Center for Disease Control and Prevention, Harbin Medical University, Harbin, Heilongjiang Province, China; 4 NHC Key Laboratory of Etiology and Epidemiology (Harbin Medical University), Harbin, Heilongjiang Province, China; 5 Joint Key Laboratory of Endemic Diseases (Harbin Medical University, Guizhou Medical University, Xi’an Jiaotong University), Harbin, Heilongjiang Province, China; 6 Key Laboratory of Heilongjiang Province for Genetically Modified Animals, Harbin Medical University, Harbin, Heilongjiang Province, China; 7 Key Laboratory of Preservation of Human Genetic Resources and Disease Control in China (Harbin Medical University), Ministry of Education, Harbin, Heilongjiang Province, China; 8 Dongzhimen Hospital, Beijing University of Chinese Medicine, Beijing, China

**Keywords:** Alzheimer’s disease, ferroptosis, molecular targeted therapy, nerve regeneration, neurodegenerative diseases, neuroinflammatory diseases, oxidative stress, Parkinson’s disease, pyroptosis, RNA methylation

## Abstract

Neurodegenerative diseases are characterized by a decline in brain structure and function. Their pathology involves multiple cell death pathways, including ferroptosis, cuproptosis, and pyroptosis. These pathways are intricately linked to genes associated with metabolism, antioxidant defense, lipid metabolism, chronic inflammation, and nerve regeneration processes. Key regulators of atypical cell death pathways show aberrant N^6^-methyladenosine modification levels under pathological conditions. As the most abundant and dynamic RNA modification in brain tissue, N^6^-methyladenosine plays crucial functional roles. Notably, there exists an intricate interplay between N^6^-methyladenosine modifications and these cell death pathways, both of which are robustly associated with the pathogenesis of neurodegenerative diseases. However, the molecular mechanisms underlying this association remain unclear. This paper reviews the correlation between N^6^-methyladenosine and various cell death patterns in neurodegenerative diseases, with emphasis on the molecular mechanisms underlying the interaction between N^6^-methyladenosine epigenetic regulation and ferroptosis, cuproptosis, and pyroptosis in cognitive impairment. N^6^-methyladenosine-modified ferroptosis plays an important role in neurodegenerative diseases. There is also a close association between N^6^-methyladenosine modification and key molecules related to cuproptosis, which may promote the deposition of copper in the brain. Chronic inflammation, a hallmark of neurodegenerative diseases, is related to pyroptosis and N^6^-methyladenosine modification. It is widely thought that ferroptosis, cuproptosis, and pyroptosis are interconnected processes that may share a common pathway affecting the pathogenesis of neurodegenerative diseases, and are related to key molecules involved in N^6^-methyladenosine epigenetic modification. This suggests a great potential for future neurodegenerative diseases treatment strategies regulated by N^6^-methyladenosine modification. N^6^-methyladenosine modification plays a dual role in nerve injury and regeneration by dynamically regulating processes such as ferroptosis, cuproptosis, and pyroptosis and their key molecules. It maintains the “death-regeneration” balance in oxidative stress and inflammation while selectively promoting axon regeneration through the modulation of methylases. This mechanism indicates a considerable therapeutic target for neurological disorders.

**Facts**

• N^6^-methyladenosine modification and neurodegenerative diseases: N^6^-methyladenosine modification is closely related to the pathogenesis of neurodegenerative diseases by influencing RNA metabolic processes such as splicing, nuclear export, translation, and degradation, thereby regulating neuronal function, oxidative stress, and neuroinflammation.

• Ferroptosis, cuproptosis, and pyroptosis: Ferroptosis is associated with disturbances in iron metabolism, lipid peroxidation, and antioxidant defense mechanisms; cuproptosis is linked to copper metabolism imbalance, mitochondrial dysfunction, and protein toxicity stress; while pyroptosis is related to neuroinflammation and immune responses.

• Relationship between N^6^-methyladenosine modification, cell death, and nerve regeneration: N^6^-methyladenosine modification influences the response of cells to injury and the various stages of nerve regeneration by regulating molecules and pathways associated with programmed cell death, ultimately determining cell fate and playing a key role in the occurrence and progression of neurodegenerative diseases.

**Open questions**

• How do N^6^-methyladenosine modifications interact with other epigenetic modifications (such as DNA methylation and histone modifications) to co-regulate the pathogenesis of neurodegenerative diseases?

• How do different programmed cell death pathways, such as ferroptosis, cuproptosis, and pyroptosis, interact with each other and achieve precise regulation for the treatment of neurodegenerative diseases?

• How can the blood–brain barrier permeability, drug delivery efficiency, and clinical safety of N^6^-methyladenosine modification-regulating drugs be improved for the treatment of neurodegenerative diseases?

## Introduction

Degenerative diseases of the nervous system impose significant medical and public health burdens on populations worldwide. Alzheimer’s disease (AD), the most prevalent neurodegenerative disorder among older individuals, is characterized by progressive memory loss, cognitive decline, and abnormal behaviors that severely impair daily functioning (Long et al., 2025). The key pathological features of AD include amyloid plaques and neurofibrillary tangles, which result from the oligomeric assembly and accumulation of amyloid-β (Aβ) peptides, along with the deposition of hyperphosphorylated tau proteins (Huang et al., 2023a). These proteins are neurotoxic and can lead to oxidative stress, mitochondrial dysfunction, inflammation, and endoplasmic reticulum stress, resulting in synaptic dysfunction and neuronal loss. This neuronal dysfunction and cell death disrupt synapses, ultimately leading to cognitive impairment in patients with neurodegenerative diseases (Zhou et al., 2023a).

N^6^-methyladenosine (m^6^A) is a reversible modification with dynamic effects on RNA functions that affects multiple RNA metabolic processes, including degradation, translation, splicing, and nuclear export (Desrosiers et al., 1974). This modification is initiated by methyltransferases, removed by demethylases, and recognized by m^6^A-binding proteins. The m^6^A “writers” consist of methyltransferase-like protein 3 (METTL3), methyltransferase-like protein 14 (METTL14), and Wilms tumor 1-associated protein, while the “erasers” include AlkB homolog 5 (ALKBH5) and fat mass and obesity-associated protein (FTO). “Readers” comprise proteins such as YTH domain-containing family members YTHDF 1, YTHDF2, YTHDF3, YTHDC1, and YTHDC2. Current evidence suggests that m^6^A is prevalent in the brain and participates in neural activities including neurogenesis, learning and memory, brain development, and axonal regeneration. The tight regulation of m^6^A in these brain functions is essential for normal brain development (Shafik et al., 2021). Emerging evidence suggests that m^6^A and its regulatory proteins are linked to numerous neurological diseases, such as AD, Parkinson’s disease (PD), depression, stroke, brain injury, epilepsy, and glioma (Han et al., 2020). In this respect, m^6^A modification may contribute to AD pathogenesis by adjusting the protein levels of disease-related genes. For example, METTL3 contributes to synaptic and memory impairment in AD by binding to miR-3968, which activates ubiquitin conjugating enzyme E2 K-dependent ubiquitination and degradation of GluN2B (Wang et al., 2023f). In addition, a genetic study with *Drosophila* models have revealed that the absence of m^6^A modification-related proteins worsens AD phenotypes and impairs motor function, indicating the essential role of m^6^A in AD (Shafik et al., 2021). Dysregulation of these signaling cascades can result in irreversible neuronal loss and dysfunction, contributing to the onset and progression of neurodegenerative diseases. Over the past 50 years, new programmed cell death (PCD) forms and their signaling pathways have been documented, including necroptosis, pyroptosis, ferroptosis, cuproptosis, mitochondrial permeability transition-induced necrosis, autophagy-dependent cell death, and lysosome-dependent cell death (Guo et al., 2024). The abnormal activation of PCD pathways is a characteristic feature of neurodegenerative diseases, and multiple dysregulated PCD signaling cascades have been observed in the pathogenesis of neurodegenerative diseases.

AD is characterized by synaptic degeneration in its early stages (Cicali et al., 2025), which precedes the onset of other destructive symptoms and is closely related to cognitive decline. Current studies have indicated that both tau protein and Aβ protein are involved in synaptic degeneration in AD (Aguado et al., 2024; Zhao et al., 2025). However, most therapies targeting Aβ and tau have not been successful (Feng et al., 2024b), leading to questions about the Aβ hypothesis as the primary pathogenic mechanism and prompting the exploration of novel mechanisms. The key pathological features of AD are senile plaques, neurofibrillary tangles, and neuronal death. Metal ions such as iron, copper, and zinc accumulate in the brain tissue of patients with AD, contributing to the development of senile plaques and neurofibrillary tangles (Faller et al., 2014). A recent study has found that specific regions in the adult brain, particularly the dentate gyrus of the hippocampus, retain the potential for neurogenesis. Interestingly, enhancing neurogenesis in the hippocampal region through exogenous transplantation or the induction of pluripotent stem cells to differentiate into neurons can improve learning and memory abilities (Wu et al., 2021). Neural stem cells can secrete neurotrophic factors such as brain-derived neurotrophic factor (BDNF) and nerve growth factor, which play a crucial role in promoting neurogenesis, maintaining synaptic connections, and facilitating synaptic formation, ultimately enhancing cognitive function (Rauti et al., 2020). However, in patients with neurodegenerative diseases, the ability for neural regeneration is greatly inhibited, which is a major factor driving the continuous deterioration of these diseases. Recent research has indicated that ferroptosis, cuproptosis, and pyroptosis possess distinct pathological features and are closely associated with the onset, progression, and prognosis of neurodegenerative diseases, offering a novel perspective for research in this field. The deposition of iron and copper induces oxidative stress, Aβ deposition, and tau protein hyperphosphorylation, which in turn leads to neuronal death. Pyroptosis intensifies neuroinflammation and impedes Aβ protein clearance, resulting in neuronal damage and death, thereby exacerbating chronic inflammation in AD. Notably, PCD is an active, genetically regulated cellular death process that serves as the “core regulator” of nerve damage, repair, and regeneration. The relationship between PCD and nerve injury, repair, and regeneration is bidirectional. It is widely thought that PCD may promote nerve repair by eliminating damaged cells and maintaining microenvironmental homeostasis, or it may exacerbate injury and inhibit regeneration due to excessive activation (Warner et al., 2023; Klemm et al., 2025). Based on this dual relationship between PCD and nerve injury, repair, and regeneration, regulating PCD has become crucial for improving neurological prognosis. By comprehensively analyzing the roles of different types of PCD in neurodegenerative diseases and through precise regulation, such as moderately inducing PCD or inhibiting excessive PCD, new targets for the clinical treatment of nerve injury are expected. In conclusion, the considerable alterations in m^6^A in the brains of patients with neurodegenerative diseases, along with its influences on genes associated with neuronal function, oxidative stress, and neuroinflammation, indicate its strong association with these diseases.

Numerous studies have shown that epigenetic modifications play a regulatory role in neural regeneration across various disease models (Shin and Cho, 2017; Gregath and Lu, 2018). However, there is still a lack of research on how m^6^A modifications regulate various PCD, balance the dynamic relationship between cell death and neural regeneration, and play pivotal role in AD. This review systematically analyzes the considerable functions of m^6^A in the above processes and innovatively proposes the m^6^A-PCD-neural regeneration regulatory axis, focusing on ferroptosis, cuproptosis, and pyroptosis. In addition, we summarize the candidate drugs targeting the m^6^A-mediated cell death axis and discuss the challenges they encounter in penetrating the blood–brain barrier, thereby enriching the potential clinical translational applications and the prospects of these drugs in AD. These findings address a gap in previous reviews, which have given insufficient attention to the interaction of various PCD pathways and their epigenetic modifications, providing a theoretical basis for clarifying the molecular regulatory network of neural regeneration and analyzing the pathogenesis of neurological diseases, while laying the foundation for the development of targeted therapeutic targets.

## Search Strategy

**Figure 1:**
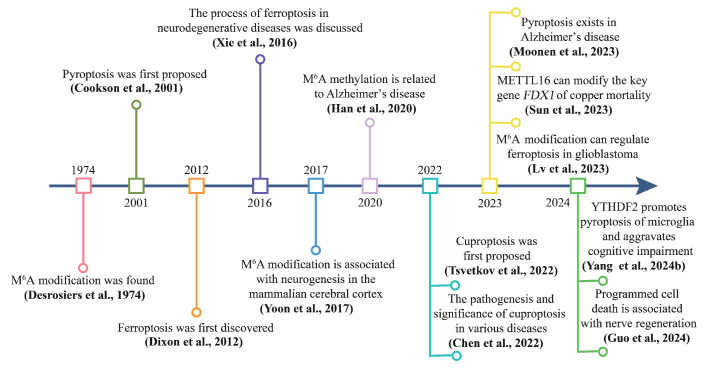
Timeline of m^6^A modification and programmed cell death in neurodegenerative diseases. FDX1: Ferredoxin 1; m^6^A: N^6^-methyladenosine.

The studies cited in this review were retrieved from the PubMed database, with most published between 2018 and 2024. Different combinations of the following keywords were used to search for existing literature: “Alzheimer’s disease,” “ferroptosis,” “Huntington’s disease,” “Parkinson’s disease,” “ amyotrophic lateral sclerosis,” “neurodegenerative diseases,” “m^6^A,” “cuproptosis,” “pyroptosis,” and “neuroregeneration.” We excluded literature that was obviously irrelevant to the research topic and conducted a preliminary screening by reading the titles and abstracts to filter out irrelevant or redundant studies. Subsequently, we performed a full-text review, ultimately including 212 articles that detailed precise mechanisms and were highly relevant to the research topic. This review provides a summary of a representative subset of the literature (**Figure 1**).

## Widespread N^6^-Methyladenosine Epigenetic Dysregulation in the Pathogenesis of Neurodegenerative Diseases

As the primary modification in eukaryotic mRNA, m^6^A modification shows considerable spatiotemporal characteristics, and its dynamic changes are closely related to the regulation of nervous system function. It is widely distributed in the brain and is involved in regulating various fundamental neurobiological processes such as neural development, axon guidance, synaptic plasticity, myelination, and stress (Shafik et al., 2021). It has been established that m^6^A methylation levels are low during early embryonic development, but they increase remarkably during adulthood as the human body matures (Han et al., 2020). In terms of spatial distribution, the regionalized regulation of m^6^A modification enables the regulation of the physiological functions of neurons in different brain regions. METTL3 is required for hippocampal-dependent memory consolidation, while METTL14 regulates transcriptional programs that are critical for striatal function and learning (Han et al., 2020). The molecular basis for this spatiotemporal specificity is that m^6^A exerts extensive control over RNA metabolism processes such as splicing, nuclear export, translation, and degradation. Abnormal m^6^A modification is associated with the onset and progression of a variety of neurodegenerative diseases. For example, abnormally elevated m^6^A levels have been observed in the cortex and hippocampus of AD models (Han et al., 2020). These abnormally modified genes are mainly enriched in synaptic function-related pathways, encoding regulators of presynaptic and postsynaptic membranes and synaptic growth (Han et al., 2020). Downregulation of FTO expression can trigger an abnormal immune response through the Notch1 signaling pathway (Qiao et al., 2024), which provides a new perspective for understanding the molecular mechanism of m^6^A modification involved in neurodegenerative diseases. From a broader disease perspective, abnormal expression of m^6^A and its regulatory proteins is also involved in various neurological diseases, including mood disorders, cerebrovascular diseases, epilepsy, and gliomas (Shafik et al., 2021), suggesting the core role of this epigenetic regulatory mechanism in the physiological and pathological processes of the nervous system.

## Programmed Cell Death in Neurodegenerative Diseases

### Role of ferroptosis in neurodegenerative diseases

#### Brain iron deposition

Under physiological conditions, the brain maintains relatively low concentrations of iron, but its spatial distribution and biological roles are crucial for proper neurological functioning. Iron is predominantly localized within hippocampal and amygdalar neurons, where it participates in metabolic activities and oxidation-reduction processes that facilitate the production of neurotransmitters and the formation of myelin sheaths. In contrast, patients with neurodegenerative diseases exhibit marked disturbances in iron regulation, leading to pathological deposition. Imaging and postmortem tissue analyses have shown considerable iron accumulation in the temporal, parietal, and frontal cortices. Notably, iron tends to concentrate around characteristic AD pathological structures such as neuritic plaques and neurofibrillary tangles (Ashraf et al., 2020; Ayton et al., 2020). In contrast to other brain regions, the cerebellum exhibits no remarkable changes in iron levels. Elevated iron deposition in the brain has been strongly linked to cognitive deterioration and the progression of neurodegenerative diseases. When iron accumulates excessively, it triggers oxidative stress, leading to DNA damage, disruption of neuronal membranes, and accelerated neurodegenerative processes (Ayton et al., 2020). One critical consequence of iron overload is ferroptosis, a distinct form of regulated cell death that involves iron-dependent lipid peroxidation. Specifically, Fe^2+^ and lipoxygenase enzymes catalyze the oxidation of membrane polyunsaturated fatty acids (PUFAs), resulting in neuronal damage and progressive cognitive dysfunction (Dixon et al., 2012). While short-term expression of heme oxygenase-1 (HO-1) may yield antioxidant and anti-inflammatory protective effects, long-term overexpression can induce tau pathology and cognitive decline through mechanisms such as iron accumulation and reactive oxygen species (ROS) generation (Wang et al., 2015). In light of these mechanisms, the contribution of iron dysregulation to the pathogenesis of neurodegenerative diseases has been well established.

It is now understood that AD progresses as Aβ peptides accumulate in the brain. Research has demonstrated that Fe^2+^ promotes AD progression by forming a stable complex with Aβ peptides and accelerating their aggregation (Tahmasebinia and Emadi, 2017). Dysregulated iron levels lead to an increase in reactive oxygen and nitrogen species, which not only worsen AD pathology but also directly attack neurons by interfering with the generation and clearance pathways of Aβ peptides (Peters et al., 2015). Fe^2+^ enhances Aβ aggregation and neurotoxicity by modulating the redox state of iron, thereby creating a vicious cycle. Additionally, Aβ peptides disrupt intracellular iron homeostasis by interfering with iron regulatory mechanisms and promoting iron reduction (Peters et al., 2015). Iron dysregulation further promotes Aβ deposition, while accumulated Aβ peptides exacerbate the dysregulation of iron levels, creating a complex interaction that collectively drives disease progression (Yuan and Gao, 2013). The above studies share a common assertion regarding the central role of iron metabolism dysregulation in the pathogenesis of neurodegenerative diseases such as AD. Based on this understanding, developing therapeutic strategies that regulate the body’s iron homeostasis is expected to achieve dual intervention effects by alleviating oxidative stress damage and inhibiting ferroptosis. This therapeutic approach offers a promising new direction for delaying the progression of neurodegenerative diseases (Ashraf et al., 2020).

#### Iron metabolism

Iron is an essential trace element involved in many key physiological processes, including oxygen transport, electron transfer, DNA repair, and gene expression regulation. The human body maintains iron homeostasis through a precise regulatory network that strictly controls the entire process of iron absorption, transport, storage, utilization, and excretion. Disruption of this balance can lead to various pathological changes. In patients with AD, iron metabolism is dysregulated, characterized by elevated ferritin levels and increased free iron content, along with a decrease in zinc content. This disorder of iron homeostasis often coexists with characteristic pathological changes, such as Aβ plaque deposition and tau protein tangles. These abnormalities can trigger considerable oxidative stress responses, leading to neuronal damage, indicating that disorders of iron metabolism may be an important driving factor for the onset and progression of neurodegenerative diseases in AD.

Dysregulation of iron metabolism in AD is closely related to alterations in multiple biomarkers and gene expression. Studies have shown a remarkable correlation between apolipoprotein E (APOE) and ferritin levels in cerebrospinal fluid (Ayton et al., 2015, 2023). In the AD Neuroimaging Initiative cohort, cerebrospinal fluid ferritin levels, which serve as markers of iron metabolism, were found to be negatively correlated with cognitive function (Pereira et al., 2024). Disorders of iron metabolism in AD can lead to excessive iron accumulation, causing ferroptosis in neurons. Recent research has found that the *Isthmin-1* gene is down-regulated in AD models, and betulin may reduce iron accumulation and affect neuronal ferroptosis through *Isthmin-1* (Bozkurt and Görücü Yılmaz, 2023; Yan et al., 2024a). Ferritin heavy chain 1 (FTH1), as a key component of ferritin, is responsible for the storage and release of iron ions. The down-regulation of FTH1 expression in the brain tissue of patients with AD exacerbates iron metabolism disorders and neuronal damage, thereby accelerating the pathological process of AD. Ferroportin 1 (Fpn1) serves as the sole iron export protein that governs iron accumulation. Studies in patients with AD and mouse models have found that the Fpn1 protein responsible for iron transport gradually decreases with age, leading to iron accumulation in the brain and subsequent cognitive decline (Xian-hui et al., 2015; Bao et al., 2024). Increasing Fpn1 expression has been shown to reduce the neural damage caused by iron deposition and even improve memory (Bao et al., 2024). Cellular iron uptake is primarily mediated by transferrin receptor 1 (TfR1), and Aβ_1–42_ interferes with this pathway, disrupting iron metabolism in synapses and triggering ferroptosis. Based on this mechanism, pre-treatment of synaptosomes with boric acid and betaine has been reported to reduce the activity of TfR1, thereby preventing ferroptosis. Additionally, the mTOR-TfR1 signaling axis is essential for maintaining iron balance, and its dysregulation may accelerate ferroptosis. Specific drugs, including spermidine and dexmedetomidine, can protect neurons and improve cognitive function by regulating this pathway (Qiao et al., 2023; Youssef et al., 2024). These findings collectively indicate that disorders of iron metabolism play a key role in the development of neurodegenerative diseases and that drugs targeting iron regulatory pathways may become a new strategy to alleviate the symptoms of neurodegenerative diseases.

Current evidence suggests that m^6^A modification regulates ferroptosis by influencing iron metabolism, lipid metabolism, and cellular defense mechanisms (**Additional Table 1**). The m^6^A modification is ubiquitous in molecules involved in brain iron metabolism and plays a key role in regulating ferroptosis. The transferrin receptor (TFRC) is a membrane-bound receptor that maintains intracellular iron homeostasis by mediating cellular iron uptake through binding to transferrin. In PC12 cells, knockdown of METTL3 reduces ferroptosis by decreasing the m^6^A modification of TFRC mRNA (Zhang et al., 2023a). Conversely, another study revealed that METTL3 overexpression can inhibit ferroptosis by upregulating the E3 ubiquitin ligase NEDD4L, stabilizing NEDD4L mRNA, and promoting TFRC ubiquitination and degradation (Su et al., 2024b). While these findings may seem contradictory, they suggest the complex relationship between m^6^A modification and iron metabolism (Zhang et al., 2023a; Su et al., 2024b). More research is warranted to explore the mechanism of action of METTL3 in different models and cell types to clarify its potential impact on neurodegenerative diseases. Additionally, the m^6^A demethylase FTO destabilizes FTH1 mRNA through m^6^A demethylation, reduces FTH1 protein expression, and inhibits ferroptosis. These findings collectively indicate that m^6^A modification regulates iron metabolism in the brain and affects ferroptosis through multiple pathways.

**Additional Table 1:** Roles of RNA m^6^A-regulated ferroptosis in neurodegenerative diseases

Disease type	Cell line	m^6^A regulator	Target	Molecular mechanism	Effects on ferroptosis	Reference
Alzheimer's disease	SH-SY5Y cells	METTL14	TUG1	Activate the GDF15/Nrf2 axis and inhibit GDF15 ubiquitination	Inhibit	Gu et al., 2024
Parkinson's disease	SH-SY5Y cells	FTO	NRF2	Reduce Nrf2 mRNA stability	Promote	Shao et al., 2024
Cerebral ischemia- reperfusion injury	HT22 cells	FTO	FYN	Inhibit FYN expression and Drp1 activity and reduce neuronal mitochondrial fission	Inhibit	Zhang and Gong, 2024
Intracerebral hemorrhage	PC12 cells	METTL3	TFRC	Increase m^6^A levels and mRNA expression of TFRC	Promote	Zhang et al., 2023a
Neonatal cerebral hypoxic ischemia	HT22 cells and BV2 cells	FTO	FTH1	Inhibit FTH1 mRNA expression and stability	Inhibit	Chen and Huang, 2024
Parkinson's disease	DA MN9D cells	METTL14	TRAF6	Downregulation of TRAF6 expression inactivates the cGAS-STING pathway and alleviates mitochondrial dysfunction	Inhibit	Shao et al., 2024
Ischemia-reperfusion injury	HT22 cells	YTHDF1	BECN1	HIF-1α binds to the promoter region of YTHDF1 to promote its transcription, and YTHDF1 stabilizes BECN1 mRNA	Inhibit	Ma et al., 2023
Intracerebral hemorrhage	BV2 cells	Not described	ctsb	Increase levels of Ctsb protein	Promote	Lu et al., 2024a
Parkinson's disease	MN9D cells	YTHDF1	ACSL4	Enhance m6A methylation in ACSL4 5'UTR and ACSL4 protein expression	Promote	Feng et al., 2024a
Glioblastoma	U87MG cells and U251 cells	NKAP	SLC7A11	Reduction of NKAP promotes SLC7A11 mRNA splicing, promotes SLC7A11 expression, and induces cell ferroptosis	Promote	Sun et al., 2022
Glioblastoma	HEK293T cells	ALKBH5, YTHDF2	GCLM	ALKBH5 regulated ferroptosis through YTHDF2-mediated decay of the GCLM	Inhibit	Lv et al., 2023
Glioblastoma	U87MG, U251, A172, LN229, U373, T98G	SND1	Nrf2	Disruption of SND1's mA-dependent recognition of Nrf2 mRNA 3'UTR reduces Nrf2 mRNA stability	Promote	Zheng et al., 2023
Glioblastoma	U87, HS683, U251, HEK 293 T	IGF2BP3	GPX4	Increase GPX4 protein expression levels	Inhibit	Deng et al., 2024
Glioblastoma	U251 cells	METTL3	FSP1	METTL3 upregulation stabilizes FSP1 mRNA	Inhibit	Bu et al., 2023
Hyperbilirubinemia	PC12 cells	ALKBH5	ACSL4	ALKBH5 promotes the stability of ACSL4 mRNA by binding to m6A modification sites within the 3' UTR of ACSL4 mRNA	Promote	Zhou et al., 2024b
Ischemic stroke	HT-22 cells and Neuro 2a cells	- FTO, YTHDF3	ACSL6	As-IV promotes the transcription of FTO by upregulating activating transcription factor 3, YTHDF3 binds to ACSL4, and reduces ACSL4 m^6^A levels	Inhibit	Jin et al., 2023
Cognitive dysfunction and depressionlike behaviors	BV2 cells	ALKBH5	PRMT2	ALKBH5 enhances the stability of PRMT2 mRNA, and PRMT2 promotes arginine methylation of β-catenin and induces proteasomal degradation of β-catenin protein, leading to transcriptional repression of GPX4	Promote	Mao et al., 2024
Cobalt-induced neurodegenerative damage	H4 cells	ALKBH5	HO-1	ALKBH5 deficiency affects the posttranscriptional regulation of HO-1, thereby affecting mRNA stability, intracellular distribution, and alternative splicing, enhancing susceptibility to Co-induced ferroptosis	Promote	Su et al., 2024a
Stroke	HT-22 cells	METTL3	NEDD4L	METTL3 can methylate and stabilize NEDD4L mRNA, enhance NEDD4L expression, ubiquitinate and degrade TFRC by NEDD4L, and reduce iron accumulation and neuronal ferroptosis	Inhibit	Su et al., 2024b

M^6^A modification regulates ferroptosis by regulating iron metabolism, lipid metabolism, and cellular defense mechanisms. ACSL4: Acyl-CoA synthetase long chain family member 4; ACSL6: acyl-CoA synthetase long chain family member 6; ALKBH5: alkB homolog 5; BECN1: beclin 1; FSP1: ferroptosis suppressor protein 1; FTH1: ferritin heavy chain 1; FTN: ferritin; FTO: fat mass and obesity-associated protein; FYN: FYN proto-oncogene, Src family tyrosine kinase; GCLM: glutamate-cysteine ligase modifier subunit; GPX4: glutathione peroxidase 4; HO-1: heme oxygenase-1; IGF2BP3: insulin-like growth factor 2 mRNA-binding protein 3; m^6^A: N^6^-methyladenosine; METTL14: methyltransferase-like 14; METTL3: methyltransferase-like 3; NEDD4L: NEDD4 like E3 ubiquitin protein ligase; NKAP: NF-kB activating protein; Nrf2: nuclear factor erythroid 2-related factor 2; PRMT2: protein arginine methyltransferase 2; SLC7A11: solute carrier family 7 member 11; SND1: staphylococcal nuclease and tudor domain containing 1; TRAF6: TNF receptor-associated factor 6; TUG1: taurine-upregulated gene 1; YTHDF1: YTH domain-containing family protein 1; YTHDF2: YTH domain-containing family protein 2; YTHDF3: YTH domain-containing family protein 3.

The iron content in the brain of patients with neurodegenerative diseases is often abnormally increased, and this dysregulation can trigger a series of chain reactions. Excess iron ions cause oxidative damage to neurons by catalyzing lipid peroxidation, activating inflammatory signaling pathways and triggering PCD (Ding et al., 2009; Raven et al., 2013). Importantly, m^6^A plays an important regulatory role in neurodegenerative diseases. Moreover, m^6^A modification can affect the maintenance of intracellular iron balance, and this regulatory effect may accelerate the progression of neurodegenerative diseases by altering ferroptosis sensitivity. The molecular interaction between m^6^A modification and iron metabolism disorders is a potential target for the development of novel neuroprotective drugs.

#### Lipid peroxidation

Cellular stress triggers sustained lipid peroxidation primarily through the action of lipoxygenases. The antioxidant defense system, comprising glutathione peroxidase 4 (GPX4) and reduced glutathione (GSH), typically converts phospholipid hydroperoxides to their corresponding alcohols while oxidizing GSH to glutathione disulfide (GSSG). However, impairment of this system through GPX4 inactivation or GSH deficiency leads to the progressive accumulation of phospholipid peroxides and the subsequent generation of lipid radicals. These reactive species form cytotoxic aldehydes that compromise membrane integrity and permeability, resulting in irreversible cell damage. Ferroptosis is now considered to depend on iron-mediated oxidative processes. Intracellular ferrous ions catalyze ROS production via the Fenton reaction. These ROS can target PUFAs within cellular membranes and induce lipid peroxidation. Lipid peroxidation is a central mechanism in ferroptosis, as it disrupts membrane structure and function, ultimately leading to cell death. Lipid peroxidation is closely associated with neurodegenerative diseases. In the brains of AD model animals, lipid peroxidation levels are remarkably increased, leading to oxidative damage to lipids and proteins, and subsequent neuronal death (Youssef et al., 2024). As a key enzyme in PUFA metabolism, acyl-CoA synthetase long-chain family member 4 (ACSL4) plays a crucial role in ferroptosis. The inhibition of ACSL4 effectively reduces the accumulation of lipid peroxides, thereby protecting neurons from ferroptosis (Bouchaoui et al., 2023). Ferroptosis, accompanied by ACSL4 upregulation, has also been observed in the hearts of amyloid precursor protein (APP)/ presenilin 1 (PS1) transgenic mice. Mitochondrial aldehyde dehydrogenase has been shown to ameliorate cardiac contractile dysfunction in APP/PS1 mice by suppressing ACSL4-dependent ferroptosis (Zhu et al., 2022). Research has also found that targeting the spermidine/spermine N1-acetyltransferase 1/arachidonic acid 15-lipoxygenase (ALOX15) signaling pathway with drugs could reduce ferroptosis in the brains of APP/PS1 mice, thereby improving cognitive dysfunction. This suggests that ALOX15 plays a major role in AD-related ferroptosis, and the spermidine/spermine N1-acetyltransferase 1/ALOX15 axis may be a new target for AD diagnosis and treatment (Ren et al., 2024; Youssef et al., 2024). Moreover, lipoxygenases, such as fatty acid synthase and ALOX5, are potential therapeutic targets (Karuppagounder et al., 2018; Ates et al., 2020). Therefore, multiple lipoxygenases play an essential role in lipid peroxidation, and targeting them can help inhibit ferroptosis and improve cognitive impairment.

Similar to its role in iron metabolism, m^6^A plays a crucial role in lipid peroxidation. Soot nanoparticles have been reported to promote ferroptosis in dopaminergic neurons by recruiting the YTHDF1 protein, enhancing m^6^A methylation in the 5′ untranslated region (UTR) of ACSL4, amplifying ACSL4 protein expression, and thus accelerating the occurrence and progression of neurodegeneration (Feng et al., 2024a). Additionally, ALKBH5 reportedly interacts with m^6^A modification sites at positions 669 and 2015 in the 3′ UTR of ACSL4 mRNA, enhancing its stability and triggering hyperbilirubinemia-induced ferroptosis (Zhou et al., 2024b).

A growing number of studies have indicated a marked increase in lipid peroxidation in neurodegenerative diseases, causing cellular membrane damage, functional deficits, and accelerated neuronal death, which worsens disease outcomes (Reichert et al., 2020; Wang et al., 2023). Given that m^6^A modification influences ferroptosis through lipoxygenase modulation, it is highly conceivable that m^6^A plays a regulatory role in these pathological mechanisms.

#### Defense pathways

The Xc-GSH-GPX4 axis serves as a crucial protective system against ferroptosis, relying on the coordinated action of several critical components, including nuclear factor erythroid 2-related factor 2 (Nrf2), GPX4, SLC7A11, and GSH. These elements work synergistically to counteract oxidative damage and suppress ferroptosis. Nrf2, a master regulator of antioxidant responses, enhances the transcription of various genes associated with glutathione production and oxidative defense mechanisms. GPX4 harnesses GSH to convert toxic lipid peroxides into harmless lipid alcohols, protecting cell membranes from oxidative destruction. Meanwhile, SLC7A11, a key subunit of the system Xc^–^ transporter, mediates cystine import in exchange for glutamate. This process is essential for maintaining intracellular GSH levels, which in turn sustains GPX4 antioxidant activity (Yang et al., 2014).

In addition to the GPX4 pathway, the FSP1/CoQ defense pathway is another critical mechanism for protecting cells against ferroptosis. Ferroptosis suppressor protein 1 (FSP1) acts as an antioxidant by reducing ubiquinone (CoQ) to ubiquinol (CoQH_2_). CoQH_2_ then neutralizes lipid peroxides, prevents the propagation of lipid peroxidation chain reactions, and prevents ferroptosis. This GPX4-independent mechanism provides cells with an alternative protective strategy against oxidative stress (Bersuker et al., 2019).

The ferroptosis defense mechanisms are severely disturbed in patients with neurodegenerative diseases, affecting key molecules greatly (Xie et al., 2016). Studies have found that Nrf2 expression is downregulated in the brain tissue of patients with AD, leading to impaired antioxidant function (Kang et al., 2024; Youssef et al., 2024). Enhancing Nrf2-mediated elimination of lipid peroxidation and strengthening the antioxidant network may help reactivate the defense system, thereby resisting ferroptosis and mitigating associated neuronal damage (Youssef et al., 2024). Additionally, the absence or mutation of the presenilin gene in patients with AD disrupts selenium uptake and inhibits the expression of GPX4, making cells more susceptible to ferroptosis and accelerating neuronal death (Greenough et al., 2022). As a GPX4 activator, tannic acid effectively inhibits ferroptosis and alleviates AD symptoms (Baruah et al., 2023). Overexpression of SLC7A11 may elevate glutamate levels, promote ferroptosis, and affect Aβ metabolism and tau protein phosphorylation levels, thereby influencing Aβ deposition and tau pathology. Beyond traditional oxidative stress regulation, the p53 protein is a crucial inducer of ferroptosis. The p53 protein reportedly regulates the expression of SLC7A11 by binding to murine double minute 2, thereby affecting the lipid peroxidation process (Yang et al., 2023b).

The ratio of GSH to GSSG is an essential cellular antioxidant indicator. In patients with AD, the ratio of intracellular glutathione to oxidized glutathione is considerably decreased, reflecting a weakening of the antioxidant defense system, which increases the susceptibility of neurons to oxidative stress and ferroptosis. This metabolic imbalance promotes Aβ plaque deposition and abnormal tau protein phosphorylation, leading to progressive neuronal loss and cognitive impairment (Gong et al., 2024). Moreover, salidroside activates the Nrf2/GPX4 pathway and enhances the expression of SLC7A11, solute carrier family 3 member 2, and GPX4, thereby inhibiting ferroptosis and reducing neuroinflammatory damage (Yang et al., 2023a). In neurons, the FSP1 enzyme converts coenzyme Q into hydroquinone, which acts as a potent lipid free radical scavenger that blocks the chain reaction of lipid peroxidation (Nelson et al., 2024; Yong et al., 2024). The FSP1/CoQ system works synergistically with the Nrf2/GPX4/GSH pathway to protect neurons and delay the progression of neurodegenerative diseases.

The m^6^A modification plays an important role in the ferroptosis defense mechanism, similar to its roles in lipid peroxidation and iron metabolism (Lv et al., 2023). In AD, METTL14-mediated m^6^A modification of TUG1 activates the Gdf15/Nrf2 signaling axis, inhibiting Gdf15 ubiquitination to stabilize Gdf15, thereby attenuating ferroptosis in AD (Gu et al., 2024). In addition to AD, m^6^A has critical regulatory functions in neurological diseases and is widely involved in neurodegenerative processes. In a PD model, FTO demethylated Nrf2 mRNA, which reduced its stability and promoted ferroptosis (Pang et al., 2024). Additionally, SNAI3-AS1 was found to competitively bind to staphylococcal nuclease domain-containing 1, interfering with the recognition of Nrf2 mRNA by staphylococcal nuclease domain-containing 1, reducing the mRNA stability of Nrf2 and promoting ferroptosis (Zheng et al., 2023). A study revealed that insulin-like growth factor 2 mRNA-binding protein 3 (IGF2BP3) increased the protein expression level of GPX4 by directly binding to a specific motif in GPX4 mRNA, thereby inhibiting glioma ferroptosis. The m^6^A modification of this motif is essential for GPX4 mRNA stability and translation (Deng et al., 2024). Nuclear Factor kappa-light-chain-enhancer of activated B cells (NF-κB) activating protein reduction promoted SLC7A11 mRNA splicing, thereby enhancing SLC7A11 expression and increasing cell sensitivity to ferroptosis inducers *in vivo* (Sun et al., 2022). Reduced NF-κB was found to promote SLC7A11 mRNA splicing, thereby upregulating SLC7A11 expression (Sun et al., 2022). Therefore, each key molecule in the Xc/GSH/GPX4 defense pathway is strictly regulated by m^6^A modification. Furthermore, the FSP1/CoQ defense pathway is also affected by m^6^A modification. METTL3 upregulation stabilizes FSP1 mRNA through m^6^A modification, promoting glioma progression by inhibiting ferroptosis.

Studies have shown that cellular defense mechanisms are impaired in neurodegenerative diseases, leading to excessive accumulation of ROS and increased ferroptosis (Baruah et al., 2023; Gong et al., 2024). The m^6^A modification has emerged as a key regulator in this process by targeting molecules in defense pathways. m^6^A modification maintains redox homeostasis by regulating the expression of defense system molecules, reducing oxidative stress and providing a therapeutic avenue for neurodegenerative diseases.

**Figure 2:**
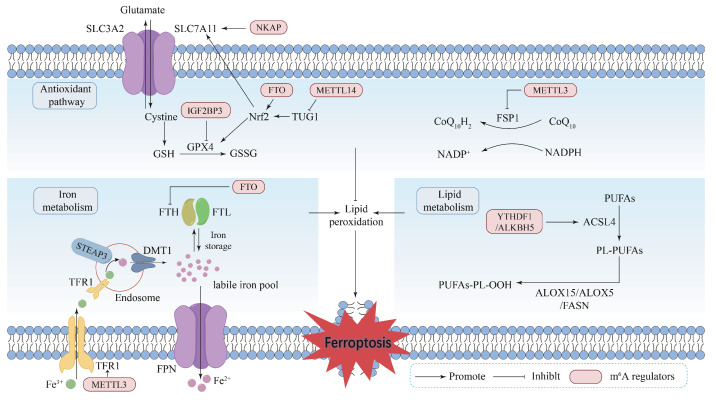
m^6^A regulates ferroptosis. m^6^A modifications remarkably impact ferroptosis by regulating lipid metabolism, iron metabolism, and antioxidant pathways, thereby influencing the progression of neurodegenerative diseases. SLC7A11 facilitates cystine uptake, supports GSH synthesis, maintains GPX4 activity, detoxifies lipid peroxides, and inhibits ferroptosis. Nrf2, a crucial transcription factor, regulates the expression of SLC7A11 and GPX4. FSP1 protects against ferroptosis by reducing CoQ to CoQH2. Dysregulated iron metabolism drives ferroptosis through mechanisms such as TFR1-mediated iron uptake, STEAP3-catalyzed iron reduction, and abnormal FTH/FTL expression. Impaired Fpn activity further increases the labile iron pool. In addition, ALOX15, ALOX5, fatty acid synthase, and ASCL4 facilitate PUFA oxidation, resulting in lipid peroxidation and neuronal damage. m^6^A modifications remarkably influence the expression and function of ferroptosis-related molecules, indirectly affecting the pathological progression of neurodegenerative diseases. ALKBH5: AlkB homolog 5; ALOX15: arachidonate 15-lipoxygenase; ALOX5: arachidonate 5-lipoxygenase; CoQ10: coenzyme Q10; CoQ10H2: ubiquinol; DMT1: divalent metal transporter 1; FASN: fatty acid synthase; FSP1: ferroptosis suppressor protein 1; FTH: ferritin heavy chain; FTL: ferritin light chain; FTO: fat mass and obesity-associated protein; GPX4: glutathione peroxidase 4; GSH: glutathione; GSSG: oxidized glutathione disulfide; IGF2BP3: insulin-like growth factor 2 mRNA-binding protein 3; m^6^A: N^6^-methyladenosine; METTL3: methyltransferase-like 3; METTL14: methyltransferase-like 14; NKAP: NF-κB activating protein; Nrf2: nuclear factor erythroid 2-related factor 2; PUFAs: polyunsaturated fatty acids; SLC3A2: solute carrier family 3 member 2; SLC7A11: solute carrier family 7 member 11; STEAP3: six-transmembrane epithelial antigen of the prostate 3; TFR1: transferrin receptor protein 1; TUG1: taurine-upregulated gene 1; YTHDF1: YTH domain-containing family protein 1.

Overall, m^6^A modification exerts multiple regulatory effects on ferroptosis, which subsequently modulates the progression of neurodegenerative diseases (**Figure 2**).

### Role of cuproptosis in neurodegenerative diseases

#### Normal physiological functions of copper

Copper is an essential trace element vital for normal cellular physiological activity, serving as a key component of nucleic acids and proteins (Zhang et al., 2023b). Under normal conditions, cellular copper metabolism is a precisely regulated process. Extracellular Cu^2+^ ions are first reduced to Cu^+^ by the enzyme six-transmembrane epithelial antigen of the prostate and then enter the cell via the copper transport protein 1. After binding to copper chaperone proteins, Cu^+^ ions are directed to specific subcellular regions, including the mitochondria, trans-Golgi network (TGN), and nucleus. In the mitochondria, Cu^+^ combines with cytochrome c oxidase to participate in the respiratory chain and redox reactions. Moreover, cytochrome oxidase 17 in the mitochondrial membrane is responsible for transferring Cu^+^ to synthesize cytochrome c oxidase 1 or cytochrome oxidase 11, which are then assembled into the cytochrome oxidase subunit. Within the nucleus, Cu^+^ binds to transcription factors and regulates gene expression. The ATPase copper transporters 7A and 7B are responsible for transporting Cu^+^ from the cytoplasm to the TGN lumen, activating copper-dependent enzymes. At elevated cytoplasmic copper concentrations, these transporters also facilitate the efflux of copper from the TGN to maintain homeostasis (Chen et al., 2022).

#### Copper metabolism and homeostasis disorder

It is now accepted that when copper ion levels exceed the homeostatic threshold, they can exert toxic effects on cells, leading to a newly identified mode of cell death known as cuproptosis. Excessive copper ions can bind to the ester acylated proteins in the tricarboxylic acid (TCA) cycle of the mitochondrial respiratory chain, resulting in the loss of fatty acylated proteins and Fe-S cluster proteins, inducing protein-toxic stress and ultimately cell death (Chen et al., 2022). There is growing evidence that copper ion deposition and imbalance are common in both patients with AD and AD mouse models (Dai et al., 2006; Miller et al., 2006; Zhang et al., 2023b). Dysregulated copper homeostasis may contribute to the pathogenesis of AD by promoting the aggregation of Aβ and tau proteins. Previous studies have shown high concentrations of copper in Aβ plaques, and that the binding of copper ions to Aβ could remarkably increase cytotoxicity (Dai et al., 2006; Miller et al., 2006). Notably, copper can also phosphorylate and aggregate tau proteins, enhancing neurotoxicity (Voss et al., 2014).

**Figure 3:**
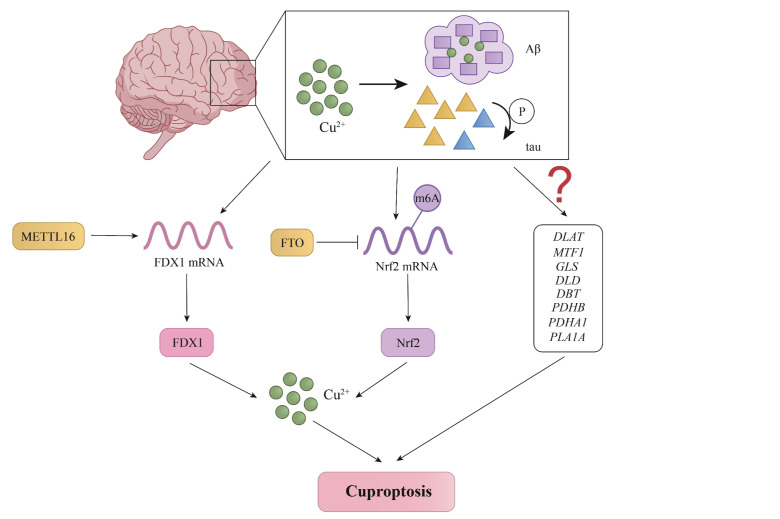
Cuproptosis and the associated mechanisms in neurodegenerative diseases. The development of neurodegenerative diseases is often accompanied by abnormal activation of cuproptosis in nerve cells, which promotes disease progression and loss of neurological function. Taking AD as an example, copper ion deposition occurs in the brains of patients with AD and AD model mice, where enhanced neurotoxicity from the aggregation of Aβ and tau may contribute to the pathogenesis of AD. The concentration of copper in Aβ plaques is high, and there is a binding of copper ions to Aβ. Furthermore, copper ions can phosphorylate and aggregate tau protein, thereby enhancing neurotoxicity. More subtle mechanisms may operate in the following ways: copper ions can promote the expression of FDX1 and Nrf2 proteins, further enhancing the accumulation of copper ions in cells and causing cell cuproptosis. This process may be related to the methyltransferases METTL16 and FTO. Additionally, data analysis results suggest that cell cuproptosis may be associated with genes such as *DLAT*, *MTF1*, *GLS*, *DLD*, *DBT*, *PDHB*, *PDHA1*, and *PLA1A*. AD: Alzheimer’s disease; Aβ: amyloid-β; DBT: dihydrolipoamide branched chain transacylase E2; DLAT: dihydrolipoamide S-acetyltransferase; DLD: dihydrolipoamide dehydrogenase; FDX1: ferredoxin 1; FTO: fat mass and obesity-associated protein; GLS: glutaminase; METTL16: methyltransferase-like protein 16; MTF1: metal regulatory transcription factor 1; Nrf2: nuclear factor E2-related factor-2; PDHA1: pyruvate dehydrogenase E1 subunit alpha 1; PDHB: pyruvate dehydrogenase E1 subunit beta; PLA1A: phospholipase A1 member A.

Current evidence suggests that copper interacts with pathogenic factors in AD, inducing neurotoxicity, oxidative stress, mitochondrial dysfunction, and disruption of lipid and glucose metabolism, ultimately leading to cognitive impairment (**Figure 3**). The core mechanism of cuproptosis involves several steps. First, Cu^2+^ ions enter the cells through SLC31A1 or copper ion carriers. Subsequently, during the mitochondrial TCA cycle, copper ions directly bind to acylated proteins, causing abnormal aggregation of these proteins (such as dihydrolipoamide S-acetyltransferase [DLAT]), resulting in the loss of Fe-S proteins through the FDX1-dependent pathway. This leads to mitochondrial dysfunction and eventually induces cell death (Tsvetkov et al., 2022; Li et al., 2025c). Studies have shown that increased brain copper levels in mice can promote the expression of apoptosis-related proteins, such as Bcl2-associated X protein (Bax), as well as cuproptosis-related proteins like ferredoxin 1 (FDX1) and DLAT. Additionally, increased copper levels can reduce Fe-S cluster proteins, inhibit the cAMP response element-binding protein (CREB)/BDNF signaling pathway, induce oxidative damage in the hippocampus, and lead to cognitive dysfunction in mice (Gale et al., 2023; Zhang et al., 2023b). Analyzing gene expression profiles of AD from comprehensive databases revealed that many cuproptosis-related genes, such as *MTF1*, *NFE2L2*, *GLS*, *DLD*, and *DBT*, could serve as markers to predict the occurrence of AD (Nie et al., 2023; Zeng et al., 2024). Least absolute shrinkage and selection operator regression analysis further indicated that ALOX5AP, PYGL, A4GALT, and PLA1A are related to cuproptosis in AD (Zhang et al., 2022a). Notably, the high expression of FDX1, DLD, GLS, PDHB, and PDHA1 was correlated with the level of Aβ_42_, which contributes to Aβ deposition and nerve damage (Lai et al., 2022; Hu et al., 2024). These key genes suggest an intrinsic relationship between copper ions and AD. Correlation analysis also showed that ALOX5AP, PYGL, A4GALT, and PLA1A participate in glucose and lipid metabolism and play essential roles in the pathogenesis of AD (Zhang et al., 2022a). Together, these results suggest the presence of abnormalities in copper metabolism in nerve cells that affect the progression of AD.

#### Regulation of cuproptosis by N^6^-methyladenosine modification

The epigenetic modification m^6^A plays an important role in neurodegenerative diseases, including the regulation of genes related to cuproptosis. Methyltransferase-like protein 16 (METTL16) is an atypical methyltransferase that regulates cuproptosis through m^6^A modification of FDX1 mRNA. High levels of copper ions can also positively interact with METTL16, inducing lactylation at K229, further enhancing cuproptosis (Sun et al., 2023). Studies have shown that YTHDF1 can also up-regulate FDX1 and promote cuproptosis in cells (Guowei et al., 2023; Zhang et al., 2023b). Additionally, the overexpression of FTO decreased the m^6^A modification of Nrf2 mRNA and enhanced the stability of Nrf2. The activation of Nrf2 reduced the oxidative stress reaction caused by copper ions. These findings demonstrate that FTO overexpression could promote oxidative stress induced by copper ions and exacerbate cognitive impairment in patients with AD (Song et al., 2014; Zhou et al., 2023b). A clinical study of patients with AD found elevated expression of PTBP1 mRNA. In mouse models, the inhibition of PTBP1 could prevent the development of AD, given that METTL3-mediated m^6^A modification enhanced the stability of PTBP1. Through ubiquitination, PTBP1 was found to promote cuproptosis and SLC31A1 expression, accelerating the progression of AD (Wang et al., 2025). An analysis of datasets of patients with epilepsy revealed that seven m^6^A-related genes (*HNRNPC*, *WATP*, *RBM15*, *YTHDC1*, *YTHDC2*, *CBLL1*, and *RBMX*) and several copper-related genes (*LIPT1*, *DLST*, and *PDHB*) were differentially expressed in both gene and m^6^A clusters. These findings highlight the potential link between m^6^A modification and copper metabolism (Liu et al., 2022).

In summary, these findings reveal a potential connection between m^6^A modification and the influence of key molecules of cuproptosis on neurodegenerative diseases, providing a new direction and strategy for exploring neurodegenerative disease therapeutics and drugs. Further research is necessary to fully elucidate the relationship between m^6^A, copper metabolism, and neurodegenerative diseases.

### Role of pyroptosis in neurodegenerative disease

#### Pyroptosis and inflammatory response

**Figure 4:**
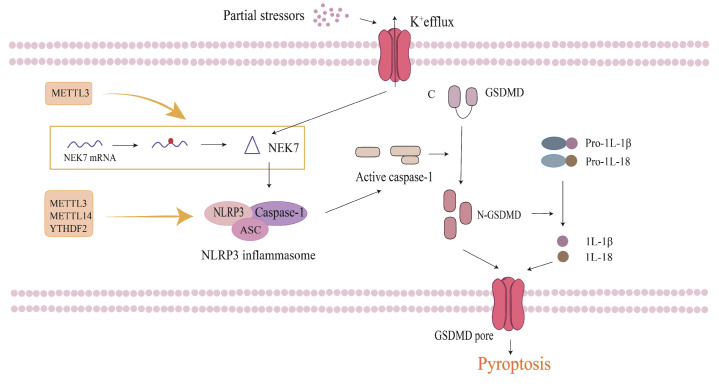
Canonical pathway of pyroptosis. In the traditional pyroptosis framework, sensor proteins of inflammatory vesicles detect cellular stress signals. These stressors indirectly activate NLRP3 through potassium efflux, facilitating NEK7 binding and triggering NLRP3 oligomerization. NLRP3 activates caspase-1 through the adaptor protein ASC. Caspase-1 activates IL-1β and IL-18, and cleaves GSDMD to release the GSDMD-N domain, which forms the membrane pore. The N-terminus integrates into the plasma membrane, oligomerizes to form a pore, and facilitates the release of activated IL-1β, IL-18, and other molecules, resulting in pro-inflammatory factor efflux, chromatin degradation, and cellular swelling. ASC: Apoptosis-associated speck-like protein containing a caspase recruitment domain; GSDMD: gasdermin D; IL-1β: interleukin-1 β; IL-18: interleukin-18; NEK7: NIMA-related kinase 7; NLRP3: NLR family pyrin domain containing 3.

In recent years, cell death in AD has been extensively studied, and in addition to ferroptosis and cuproptosis, pyroptosis has become a research hotspot. Pyroptosis is a form of inflammatory cell death that plays a crucial role in host defense against pathogenic infections by inducing a strong inflammatory response (Cookson and Brennan, 2001). This process is primarily triggered by various inflammatory vesicles and is mediated by caspases and gasdermin proteins (Hsu et al., 2021). The signaling cascade of pyroptosis encompasses classical, non-canonical, caspase-3-induced, caspase-8-triggered, and granzyme-mediated pathways. In the classical model, inflammatory vesicle-associated sensor proteins detect cellular stressors, such as bacteria, viruses, toxins, adenosine triphosphate (ATP), uric acid crystals, silica particles, as well as damage-associated molecular signatures. These stressors result in the indirect activation of the NLR family pyrin domain-containing protein 3 (NLRP3) following potassium efflux, which subsequently enables NIMA-related kinase 7 (NEK7) to bind with NLRP3 and initiate its oligomerization process. The bridging protein apoptosis-associated speck-like protein containing a caspase recruitment domain (ASC) then facilitates the activation of caspase-1 by NLRP3. Caspase-1 catalyzes the activation of interleukin-1 β (IL-1β) and interleukin-18 (IL-18), and further cleaves gasdermin D (GSDMD), leading to the release of the GSDMD-N domain, which forms pores in the membrane. The N-terminal portion inserts into the plasma membrane, undergoing oligomerization to create a pore with an approximate inner diameter of 12–14 nm. This process results in the efflux of pro-inflammatory factors, degradation of chromatin, and subsequent cellular swelling. The formation of the GSDMD-N pore allows the outward flow of activated IL-1β and IL-18, together with other cellular components (Frank and Vince, 2019), potentially inducing harm to adjacent cells and enhancing inflammatory responses (**Figure 4**). Notably, activated caspase-11, caspase-4, and caspase-5 can be integrated into NLRP3 inflammatory vesicles. This process triggers cellular pyroptosis by cleaving GSDMD, which ultimately results in the outflow of potassium ions (Guo et al., 2024). In non-canonical pathways, cytoplasmic lipopolysaccharide (LPS) activates caspases-4/5 and -11, triggering pyroptosis through the cleavage of GSDMD. However, oxPAPC competes with LPS for binding to caspases-4/1, thereby exhibiting an inhibitory effect on pyroptosis. Besides, GSDMD cleavage induces potassium ion efflux, ultimately mediating NLRP3 inflammasome assembly and leading to the cleavage of pro-IL-1β and pro-IL-18. Activated caspase-11 also cleaves pannexin-1, promoting the release of ATP and P2X7R-associated pyroptotic cell death. In the caspase-3-mediated pathway, active caspase-3 cleaves GSDME to form N-GSDME, thus inducing pyroptosis. In the caspase-8-mediated pathway, inhibition of TAK1 results in the activation of caspase-8, which cleaves GSDMD and induces pyroptosis. Furthermore, under hypoxic conditions, PDL1 translocates to the nucleus and co-regulates GSDMC transcription with p-Stat3, converting apoptosis to pyroptosis upon tumor necrosis factor-alpha (TNF-α)-activated caspase-8 action. In the granzyme-mediated pathway, CAR T cells rapidly activate caspase-3 in target cells via GzmB release, followed by GSDME activation to induce extensive pyroptosis (Wei et al., 2022). Besides, granzyme A and granzyme B in cytotoxic lymphocytes enter target cells through perforin and induce pyroptosis: Granzyme A hydrolyzes GSDMB, while granzyme B directly activates GSDME. Inflammatory cell death, specifically cellular pyroptosis, is triggered by GSDMD-mediated cell cleavage and involves inflammation-associated caspase enzymes. The formation of inflammatory vesicles initiates the activation of caspases, leading to the cleavage of gasdermin proteins into toxic fragments, which facilitate the perforation of the cell membrane. When pyroptosis leads to cell membrane disruption, cytokines such as IL-18, IL-1β, and other molecules are released, resulting in increased tissue damage (Vasudevan et al., 2023).

#### Pyroptosis and neurodegenerative diseases

In physiological processes, a moderate amount of pyroptosis plays a vital role in host defense against pathogenic microorganisms. However, dysregulated inflammatory responses and cell death caused by excessively activated pyroptosis may contribute to abnormal pathological progression of diseases, particularly inflammatory diseases, including cardiovascular diseases, liver diseases, and neurological disorders (Yu et al., 2021; Moonen et al., 2023). It is well established that the primary pathological indicators of AD are amyloid plaques and neurofibrillary tangles, arising from the oligomerization and accumulation of Aβ peptides, as well as the deposition of tau proteins that have undergone hyperphosphorylation. Both proteins exhibit neurotoxicity, which triggers oxidative stress, disrupts mitochondrial function, elicits inflammation, and induces endoplasmic reticulum stress, ultimately leading to synaptic impairment and neuronal degeneration, thus contributing to cognitive decline in patients with AD. Studies have demonstrated that patients with AD exhibit increased expression of NLRP3 and NLRP1 in brain tissue and heightened levels of GSDMD in cerebrospinal fluid. A correlation analysis revealed a link between the cerebrospinal fluid levels of GSDMD in patients with AD and markers such as T-Tau, Tau181, and Aβ_1–42_ (Shen et al., 2021). Importantly, Aβ_1–42_ can induce a notable increase in GSDMD expression within OLN-93 cells. Meanwhile, Aβ protein was found to activate NLRP3 inflammatory vesicles and drive pyroptosis (Cai et al., 2021). This suggests that Aβ_1–42_ can trigger cellular pyroptosis and can be harnessed as a relevant model for studying cellular pyroptosis in AD research (Song et al., 2024). Research has shown that 1,7-diphenyl-4-hepten-3-one could mitigate oxidative stress-induced damage and reduce the abundance of juxtaposition-related proteins, thereby potentially inhibiting cellular juxtaposition through activation of the Nrf2 signaling pathway. C1 also inhibited oxidative stress damage and reduced the levels of pro-cytokinetic proteins, which could inhibit cellular cytokines through activation of the Nrf2 pathway and the related proteins NLRP3, GSDMD, and caspase-1, as well as a reduction in the secretion of the pro-inflammatory molecules IL-1 and IL-18 (Shi et al., 2024). These findings collectively suggest the presence of abnormal cellular cytokines in patients with AD which play pivotal roles in disease progression.

#### Regulation of pyroptosis by N^6^-methyladenosine modification

The m^6^A modification is a common epigenetic process that regulates AD progression by influencing cell death pathways. Pyroptosis typically involves five key inflammatory vesicles: NLRP3, AIM2, NLRP1, PYRIN, and NLRC4. These inflammatory vesicles activate the caspase family (such as caspase-1 and caspase-3) and the gasdermin family (e.g., GSDMD), thereby inducing pyroptosis (Zhou et al., 2024c). A recent study has shown that m^6^A methyltransferase families are involved in cell death in various brain-related diseases. METTL14 enhances M1 polarization and triggers the NLRP3 inflammasome in ischemic stroke via KAT3B-STING signaling (Li et al., 2023c). Additionally, METTL14 can alleviate the stability of NLRP3 mRNA, thereby promoting pyroptosis in neuronal cells during brain hemorrhage (Li et al., 2024b). These findings suggest that targeting METTL14 could be a viable strategy to mitigate neuronal pyroptosis in the brain. The methyltransferase METTL3 also plays a role in regulating neuronal cell death. METTL3 enhances hemin-induced pyroptosis in HT-22 cells by methylating NEK7, which is recognized by IGF2BP2, thus advancing cerebral hemorrhage progression (Hong et al., 2024). Conversely, Emodin induces cell death in lipopolysaccharide-treated human astrocytes by decreasing METTL3-mediated m^6^A modification of NLRP3 mRNA, thereby inhibiting inflammation and cell death (Wang et al., 2022). Furthermore, homocysteine-induced cognitive deficits can be reduced by betaine, which attenuates NLRP3-mediated microglial cell death in an m^6^A-YTHDF2-independent manner (Yang et al., 2024b). Collectively, these findings indicate that various m^6^A regulators may influence cell death by modulating NLRP3. Cellular pyroptosis is primarily driven by the NLRP3/caspase-1/GSDMD pathway. The m^6^A regulators can also modulate cellular pyroptosis by regulating mRNAs that are indirectly related to this pathway. In ischemic stroke, reduced FTO modulates lncRNA MEG3 stability through m^6^A dependence, triggering neuronal cell pyroptosis via the NLRP3/caspase-1/GSDMD pathway (Yan et al., 2024b). The YTHDF1 protein activates sevoflurane-induced neuronal pyroptosis and cognitive dysfunction via the CREB-BDNF signaling pathway (Huang et al., 2023b). Recent studies have elucidated the role of PTEN in regulating focal cell death in human diseases (Sinha et al., 2019; Nisha et al., 2025). PTEN mRNA, which contains an m^6^A site, plays a role in low temperature-activated PI3K/Akt/GSK-3β signaling, leading to the downregulation of pyroptosis-associated proteins NLRP3 and ASC and decreased pro-inflammatory cytokine secretion, thereby protecting hippocampal neurons from hypoxia/re-oxidation-induced pyroptosis (Diao et al., 2020). Neural stem cell–derived extracellular vesicles containing Y-box binding protein 1 (YBX1) enhance the stability and expression of m^6^A -modified G protein-coupled receptor 30 (GPR30) through their interaction with IGF2BP1. The resulting upregulation of GPR30 reduces neuronal focal cell death in ischemic stroke by facilitating the ubiquitinated degradation of NLRP3 via speckle-type POZ protein, thereby inhibiting the activation of NLRP3 inflammatory vesicles (Peng et al., 2023). Overall, the above studies establish an essential bridge between m^6^A and juxtamembrane-related components, suggesting that m^6^A modification plays an important role by directly or indirectly affecting key factors such as NLRP3 mRNA, lncRNA MEG3, and NEK7 mRNA (**Additional Table 2**). As shown in **Figure 4**, it can be inferred that among the regulatory factors involved in m^6^A modification, methyltransferases METTL3 and METTL14 play a key role. This finding suggests that METTL3 and METTL14 could be therapeutic targets for targeting pyroptosis in AD. However, further research is warranted to investigate the involvement of m^6^A modifications in AD-related pyroptosis.

**Additional Table 2:** Role of m^6^A-regulated pyroptosis in neurodegenerative diseases

Disease type	Cell line	m^6^A regulator	Target	Molecular mechanism	Effects on pyroptosis	Reference
Ischemic stroke	HAPI cells	METTL14	KAT3B mRNA	Enhance KAT3B expression by promoting its m6A modification.	Promote	Li et al., 2023c
Cerebral ischemia/ reperfusion injury	Primary hippocampal neurons	No study	PTEN mRNA	Hypothermia activates PI3K/Akt signaling via m^6^A modification of PTEN mRNA.	Inhibit	Diao et al., 2020
Sevoflurane-induced neuronal pyroptosis and cognitive dysfunction	Primary hippocampal neurons	YTHDF1	CREB mRNA	Activate CREB-BDNF signaling	Promote	Huang et al., 2023b
Cerebral ischemic stroke	Primary cortical neurons and N2a cells	FTO	MEG3 mRNA	Regulate MEG3 expression through m6A modification by affecting its stability, and activating neuronal pyroptosis via NLRP3/caspase-1/GSDMD signaling.	Promote	Yan et al., 2024b
Cognitive impairment induced by homocysteine	Microglia	YTHDF2	NLRP3 mRNA	Attenuate NLRP3 mRNA stability in a YTHDF2-dependent manner	Promote	Yang et al., 2024b
Sepsis brain injury	1321N1 cells	METTL3	NLRP3 mRNA	Emodin upregulates METTL3 expression levels and promotes the m^6^A methylation modification-induced downregulation of NLRP3 in LPS-treated 1321N1 cells	Inhibit	Wang et al., 2022
Intracerebral hemorrhage	HT-22 cells	METTL3	NEK7 mRNA	The silence of METTL3 inhibits hemin-induced HT-22 cell pyroptosis via suppressing m^6^A methylation of NEK7 in an IGF2BP2- dependent manner	Promote	Hong et al., 2024
Intracerebral hemorrhage Ischemic stroke	PC-12 cells Neural stem cells	METTL14 IGF2BP1	NLRP3 mRNA GPR30 mRNA	Attenuate NLRP3 mRNA stability NSC-derived EVs carrying YBX1 increases the stability of GPR30 mRNA by interacting with IGF2BP1, and the upregulation of GPR30 inhibits the activation of NLRP3 infammasome through promoting NLRP3 ubiquitination by SPOP	Promote Inhibit	Li et al., 2024b Peng et al., 2023

m^6^A modification plays an important role in pyroptosis by directly or indirectly affecting key factors such as NLRP3 mRNA, lncRNAMEG3, and NEK7 mRNA. Akt: Protein kinase B; BDNF: brain-derived neurotrophic factor; CREB : cAMP response element-binding protein; EV: extracellular vesicle; FTO: fat mass and obesity-associated protein; GPR30:G protein-coupled receptor 30; GSDMD: gasdermin D; IGF2BP1: insulin-like growth factor 2 mRNA-binding protein 1; KAT: lysine acetyltransferases; LPS: lipopolysaccharide; MEG3: maternally expressed gene 3; METTL3: methyltransferase-like protein 3; METTL14: methyltransferase-like protein 14; m^6^A: N^6^-methyladenosine; NEK7: NIMA-related kinase 7; NLRP3: NLR family pyrin structural domain 3; NSC: meural stem cell; PI3K : phosphatidylinositol 3-kinase; PTEN : phosphatase and tensin homolog; SPOP: speckle-type POZ protein; YBX1: Y-box binding protein 1; YTHDF: YTH domain-containing family.

## Crosstalk between Ferroptosis, Cuproptosis, and Pyroptosis

### Crosstalk between ferroptosis and cuproptosis

#### Key regulatory role of FDX1

The process of cell death involves complex interactions between ferroptosis and cuproptosis. In the brains of patients with AD, iron and copper co-accumulate, particularly in Aβ plaques and neurofibrillary tangles. Both iron and copper overload can induce mitochondrial dysfunction and oxidative stress, thereby accelerating neuronal damage, which can be explained from the perspective of mitochondrial dysfunction, oxidative stress, and substance metabolism. Elevated brain copper levels can diminish Fe-S cluster proteins by enhancing the expression of apoptosis-related protein Bax and cuproptosis-related proteins FDX1 and DLAT, which worsen mitochondrial dysfunction and oxidative stress, ultimately causing cuproptosis (Zhang et al., 2023b). FDX1, which plays a role in cuproptosis, also influences ferroptosis. Specifically, FDX1 can directly induce copper ion deposition, while copper can mediate GPX4 degradation and enhance ferroptosis sensitivity (Song et al., 2025). This atypical methyltransferase METTL16 can modify FDX1 mRNA, which is a key step in copper deposition (Sun et al., 2023), highlighting the dual role of FDX1 in regulating cellular ferroptosis and cuproptosis, as well as the critical role of m^6^A modification.

#### Key regulatory role of Nrf2

Nrf2 stands as another pivotal molecule implicated in the cellular processes of ferroptosis and cuproptosis. Copper dysregulates the CREB/BDNF signaling pathway by inhibiting the phosphorylation of CREB, thereby precipitating oxidative damage within cells. This perturbation not only attenuates neuroprotective signaling cascades, inducing apoptotic pathways, but also paradoxically upregulates the Nrf2-mediated antioxidant defense system, including downstream effectors HO-1 and NQO1 (Lu et al., 2022). Activation of the Nrf2/HO-1/NQO1 pathway influences multiple facets of cellular health, from ferroptosis resistance and mitochondrial function to viability under stress conditions (Wang et al., 2023e). Moreover, FTO-mediated m^6^A demethylation of Nrf2 mRNA disrupts iron/copper metabolic regulation, suggesting a plausible mechanistic link to the pathogenesis of AD (Zhou et al., 2023b).

#### Interplay of iron and copper metabolism

Both iron and copper have been demonstrated to exacerbate the depletion of GSH and precipitate cell death, thereby indicating their potential contribution to nerve cell injury through GSH modulation. Notably, copper further compounds this effect by suppressing the activity of glutamate cysteine ligase, the rate-limiting enzyme essential for GSH synthesis, thereby hindering cellular capacity to restore its antioxidant defenses. The accumulation of these metals, iron and copper, is widely thought to play a significant role in the initiation and progression of neurodegenerative diseases, presenting a promising therapeutic approach for mitigating their detrimental effects (Maher, 2018).

Copper ions play an important role in the induction of ferroptosis by inducing autophagic degradation of GPX4 protein. Exogenous copper ions specifically bind to the cysteine residues at positions C107 and C148 of the GPX4 protein, thereby promoting its abnormal aggregation and subsequent degradation (Xue et al., 2023). This mechanism elucidates how abnormal copper ion levels promote ferroptosis. Besides, another approach has been identified to regulate GPX4. At the post-transcriptional regulatory level, the m^6^A reader protein IGF2BP3 regulates GPX4 protein expression by recognizing specific m^6^A modification sites on the GPX4 mRNA, which enhances the stability of GPX4 mRNA and translation efficiency, ultimately determining whether the cell is prone to ferroptosis (Deng et al., 2024). These findings not only reveal how copper ions and RNA modifications regulate ferroptosis, but also reveal the intrinsic connection between distinct PCD pathways, providing important clues for the future development of new therapies for neurodegenerative diseases.

Recent studies have demonstrated a relationship between lipid metabolism and iron/copper metabolism (Behl et al., 2022; Shi et al., 2023). Excessive iron promotes lipid peroxide accumulation in cell membranes through multiple metabolic pathways, including fatty acid metabolism, the mevalonate pathway, and mitochondrial respiration. This process ultimately leads to PCD (Zheng and Conrad, 2020). Least absolute shrinkage and selection operator regression analysis revealed four cuproptosis-related genes - *ALOX5AP*, *PYGL*, *A4GALT*, and *PLA1A* - that significantly influence glucose and lipid metabolism in AD (Zhang et al., 2022a). The demethylase FTO exerts regulatory effects on lipid metabolism through multiple mechanisms. It controls the expression of lipid-related genes by modulating mRNA processing, transcript maturation, and translational efficiency of critical metabolic regulators (Yang et al., 2022b). The above findings suggest that iron and copper accumulation in AD may be closely associated with dysregulated lipid metabolism. Furthermore, m^6^A epigenetic modification plays a regulatory role in this process. These insights provide therapeutic targets and directions for future research in AD.

While distinct mechanisms initiate various forms of cell death, these pathways share similarities and can either independently or concurrently be activated depending on cellular conditions. Ferroptosis and cuproptosis are interconnected and mutually influential processes. Our findings also highlight the significant role of m^6^A RNA methylation in modulating the critical molecules involved in both processes. Given the established dysregulation of metal metabolism in neurodegenerative diseases, we propose that m^6^A methylation promotes the pathogenesis of AD by disrupting metal ion balance by regulating key molecules or pathways of cellular iron and copper metabolism.

### Crosstalk between ferroptosis and pyroptosis

Ferroptosis and pyroptosis are two distinct modes of PCD that regulate neuronal cell death in AD. However, they share similarities, and examining these similarities can improve current understanding of cell death mechanisms and their roles in AD. Previous studies have shown extensive crosstalk between key pathways and molecules that mediate ferroptosis and pyroptosis in AD (Qiu et al., 2023; Balusu and De Strooper, 2024). This interconnection is evident in their shared regulation of inflammatory responses and oxidative damage. For instance, the neuroprotective effect of Rhodopsin against Aβ_25–35_-induced PC12 cytotoxicity involves the Nrf2/GPX4 and TLR4/p-NF-κB/NLRP3 pathways. Additionally, compound C1 mitigates cellular juxtaposition by activating the Nrf2 pathway, inhibiting the proteins NLRP3, GSDMD, and caspase-1, and reducing the secretion of pro-inflammatory cytokines IL-1 and IL-18 (Xia et al., 2024). Research has indicated that the m^6^A demethylase FTO mitigates neuronal apoptosis caused by oxygen-glucose deprivation/reoxygenation by decreasing m^6^A modification on Nrf2 mRNA. This suggests the significant role of FTO in ferroptosis and focal cell death in AD through Nrf2 modification (Huang et al., 2023b). In addition, M1 macrophages/microglia can affect neurons by influencing ferroptosis and pyroptosis (Li et al., 2024a). Intriguingly, M1 macrophages/microglia can reportedly induce neuronal pyroptosis, a process attenuated by quercetin, which mitigates neuroinflammation by inducing M1–M2 polarization and inhibiting neuronal pyroptosis, as well as protecting neurons from ferroptosis (Li et al., 2024a). The state of M1 macrophages/microglia is widely thought to mediate the communication between these two cell death pathways. When M1 macrophages/microglia are susceptible to ferroptosis, this state can be transferred to neurons. Conversely, when M1 macrophages/microglia are resistant to RSL3- or erastin-induced ferroptosis, pro-inflammatory cytokine levels remain high, thereby triggering pyroptosis of neurons. This suggests that M1 macrophages/microglia play important roles in the link between ferroptosis and pyroptosis. Moreover, ferroptotic damage initiates neuroinflammation through the stimulator of interferon genes (STING)-mediated DNA sensing pathway. It has been established that during pyroptosis, the activation of the STING pathway promotes the formation of inflammatory vesicles and the onset of cellular pyroptosis. Ferroptosis-induced damage results in oxidized DNA accumulation and release into the cytoplasm, activating the DNA-sensing protein STING. This activation triggers interferon-beta and other inflammatory pathways, such as NF-κB/TNFα and NLRP3/caspase1/IL-1β, the latter being recognized as a classical pathway in neuronal cell pyroptosis (Liu et al., 2023). The methylase METTL14, a key regulator of m^6^A modification, enhances KAT3B expression, which in turn promotes M1 polarization and the NLRP3 inflammasome/pyroptosis axis via KAT3B-STING signaling (Li et al., 2023c). These results suggest that implementing therapy aimed at downregulating ferroptosis injury and its downstream STING-mediated neuroinflammation could have a significant neuroprotective impact on the amyloid-induced AD model. Edaravone has been shown to decrease Aβ_1–42_-induced apoptosis and the production of pro-inflammatory factors TNF-α, IL-1β, and IL-6 in HT22 cells. It also inhibits the TLR4/NF-κB/NLRP3 signaling pathway, ferroptosis, and lipid peroxidation (Guo et al., 2023b). Inhibition of the NLRP3 inflammasome and subsequent reduction of IL-1β production contributes to the protection of HT22 cells from pyroptosis-induced damage. The ion channel TRPV4 promotes CamkIIα phosphorylation, leading to an increase in NF-κB-mediated hippocampal inflammatory vesicles and causing neuronal pyroptosis (Guo et al., 2023a). These findings collectively indicate that ferroptosis-associated molecules can exacerbate cellular pyroptosis by promoting inflammatory responses and that these molecules are also regulated by m^6^A.

GPX4 serves as a crucial regulator that links ferroptosis and pyroptosis through its role in redox regulation. As a central antioxidant enzyme preventing iron deposition, GPX4 uses GSH to detoxify lipid peroxides into lipid alcohols, thereby maintaining membrane integrity and resisting oxidative attacks (Yang et al., 2014). The protective function of glutathione extends to the regulation of pyroptosis. Supplementation with GSH ethyl ester has been shown to reverse mitochondrial GSH depletion, reduce Aβ-induced oxidative stress, and inhibit the activation of inflammasomes in neurons (de Dios et al., 2023). Mechanistically, lipid peroxidation, a hallmark of ferroptosis, initiates a series of events leading to pyroptotic cell death. The excessive accumulation of lipid ROS facilitates the assembly of the NLRP3 inflammasome, which activates caspase-1 to process the pro-inflammatory cytokines IL-1β and IL-18 while cleaving GSDMD to trigger pyroptosis (Yang et al., 2024b).

### Crosstalk between ferroptosis, cuproptosis, and pyroptosis

**Figure 5:**
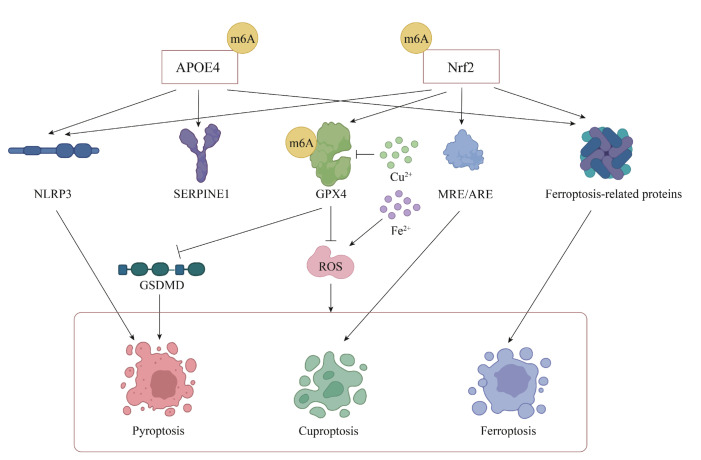
Regulation of m6A modification affects ferroptosis, cuproptosis, and pyroptosis simultaneously. M^6^A modification plays an important role in regulating key molecules such as APOE4, Nrf2, and GPX4. These molecules affect multiple cell death pathways, including ferroptosis, cuproptosis, and pyroptosis. The m^6^A modification of APOE4 influences the expression of NLRP3, SERPINE1, and proteins related to ferroptosis. Nrf2, as a central regulator, modulates proteins associated with ferroptosis, including those in the MRE/ARE pathway, NLRP3, and GPX4. Among these, GPX4 acts as a protective factor by reducing ROS accumulation and preventing GSDMD cleavage. Cu^2+^ directly inhibits GPX4 activity, while Fe^2+^ enhances ROS generation. These interrelated programmed cell death pathways share key regulatory molecules, forming a complex network. m^6^A-regulated key molecules exhibit a wide range of effects on different programmed cell deaths. APOE4: Apolipoprotein E4; ARE: antioxidant response element; GSDMD: gasdermin D; GPX4: glutathione peroxidase 4; m^6^A: N^6^-methyladenosine; MRE: metal-responsive element; NLRP3: NLR family pyrin domain containing 3; Nrf2: nuclear factor erythroid 2-related factor 2; ROS: reactive oxygen species; SERPINE1: serpin family E member 1.

Over the past decade, regulated cell death pathways, including ferroptosis, cuproptosis, and pyroptosis, have been increasingly recognized as key contributors to the pathogenesis of neurodegenerative disorders such as AD and PD. While these regulated cell death mechanisms exhibit distinct morphological features and molecular pathways, their interrelationships are complex. This review systematically examines the mutual influences and specific roles of these various cell death pathways in neurodegenerative diseases, emphasizing the potential for m^6^A RNA methylation to serve as a common regulatory node linking these pathways (**Figure 5**).

Genetic mutations have been established as key triggers for various forms of cell death. The *APOE ε4* allele has been closely associated with ferritin levels in cerebrospinal fluid. Ferritin levels serve as a biomarker that is inversely correlated with cognitive performance, with elevated concentrations indicating diminished cognitive function (Pereira et al., 2024). Interestingly, single-cell sequencing analysis revealed that inhibitory neurons in *APOE ε4* carriers are more susceptible to ferroptosis (Brase et al., 2023). In addition to its role in iron metabolism, APOE can also regulate neuroinflammation by activating the NLRP3 inflammasome in macrophages, thereby promoting the secretion of IL-1β in a concentration-dependent manner.

Gene mutations can also simultaneously trigger multiple cell death pathways. In addition to the role in ferroptosis, APOE has been shown to activate the NF-κB signaling pathway in BV2 microglial cells, which enhances NLRP3 inflammasome-mediated IL-1β secretion, and disrupts mitosis (Liu et al., 2024). The above-mentioned study suggests that APOE may be a therapeutic target for alleviating neuronal pyroptosis in neurodegenerative diseases. The regulatory role of APOE also involves cuproptosis, as evidenced by its positive correlation with *SERPINE1* expression. As a factor linked to cuproptosis, SERPINE1 exhibits negative associations with cuproptosis-related genes such as *FDX1*, *LIAS*, *LIPT1*, and *PDHA1* (Feng et al., 2023; Chen et al., 2024a, b). While these observations strongly implicate APOE in cuproptosis modulation, the underlying molecular mechanisms remain to be fully elucidated, highlighting an important area for future research.

Multiple cell death pathways can be regulated by identical key molecules. Nrf2 is a core regulatory factor involved in ferroptosis, cuproptosis, and pyroptosis. In ferroptosis, Nrf2 activates the expression of downstream antioxidant enzymes and iron transporter genes, which removes ROS and lipid peroxides, maintains intracellular iron homeostasis, and ultimately inhibits ferroptosis (Li et al., 2023a). In cuproptosis, Nrf2 binds to metal response and antioxidant response elements to activate antioxidant and detoxification genes, protecting cells from oxidative damage (Song et al., 2014). For pyroptosis, Nrf2 can suppress NF-κB signaling and modulate miRNA expression, thereby inhibiting the activation of NLRP3 inflammasome (Tufekci et al., 2021).

Importantly, one mode of cell death can influence another. Under oxidative stress conditions, Fe^2+^ interacts with hydrogen peroxide to produce ROS via the Fenton reaction. This process not only promotes ferroptosis but also affects cuproptosis through the accumulation of metal ions. The GPX4/GSH defense system protects against the Fenton reaction. Cu^2+^ can promote ferroptosis by inducing GPX4 degradation (Xue et al., 2023). Additionally, GPX4 inhibits pyroptosis by preventing GSDMD cleavage and exerting antioxidant effects. Nrf2 activation can upregulate GPX4 expression, and drugs can inhibit pyroptosis by activating the Nrf2/GPX4 pathway (Lu et al., 2024b). The link between glucose metabolism, ferroptosis, and pyroptosis has been explored in a study by Han et al. (2023). In cuproptosis, intracellular copper targets and binds to acylated components of the TCA cycle. The accumulation of copper-bound acylated mitochondrial proteins and the resulting decrease in Fe-S clusters cause proteotoxic stress and subsequent cell death (Chen et al., 2022). Therefore, glucose metabolism plays a pivotal role in ferroptosis, cuproptosis, and pyroptosis. Research has also indicated that Cu^2+^ can increase GPX4 ubiquitination, promoting pyroptosis by enhancing its interaction with the autophagy receptor Tax1 binding protein 1. Disorders in copper metabolism can also lead to ferroptosis, which may, in turn, affect copper metabolism and pyroptosis (Wang et al., 2023d). In summary, one mode of cell death can regulate others through oxidative stress, glucose metabolism, and ion effects.

Finally, ferroptosis, cuproptosis, and pyroptosis may act synergistically during disease progression. In AD, the dysregulation of iron and copper metabolism initiates interactions among various cell death pathways, thereby accelerating disease progression (Wang et al., 2023d).

Since different cell death modes share key molecular pathways, m^6^A modifications can affect various forms of cell death. FTO, an m^6^A demethylase, mitigates neuronal apoptosis by decreasing m^6^A modifications on Nrf2 mRNA. This indicates that FTO remarkably influences ferroptosis, cuproptosis, and pyroptosis in AD through Nrf2 modification (Huang et al., 2023b). Moreover, m^6^A regulates APOE expression and affects the risk of AD (Watts et al., 2022). The m^6^A reader protein IGF2BP3 further modulates cell death by directly binding to a specific motif on GPX4 mRNA, thereby regulating GPX4 protein expression (Deng et al., 2024).

The cell death pathways, ferroptosis, cuproptosis and pyroptosis, constitute a complex cross-regulatory network, which contribute to the occurrence and development of diseases. In the context of neurodegenerative diseases, an imbalance in iron and copper metabolism creates a pathological cascade, where these distinct cell death mechanisms mutually amplify each other and drive disease progression through synergistic interactions. Our findings indicate that m^6^A modification can influence core molecules, signaling pathways, and underlying mechanisms. Based upon the above findings, it can be inferred that m^6^A modification acts as a key regulator, influencing these cell death pathways by modulating key molecules and pathways, thereby affecting cell survival and death in neurodegenerative diseases.

### Immune microenvironment related to ferroptosis, cuproptosis, and pyroptosis in neurodegenerative diseases

In neurodegenerative diseases, the immunological environment primarily consists of innate immune cells, such as microglia, residing in the central nervous system, along with peripheral immune cells, including T and B lymphocytes. These immune cells secrete cytokines, chemokines, and various other immune-related substances, which interact with each other to form a complex “network.” This network not only regulates the process of neuroinflammation but also exerts a neuroprotective effect (Sabatino et al., 2019; Chen et al., 2023; Gao et al., 2023). Within the central nervous system, microglia play a dual role: on one hand, they provide neuroprotective benefits by clearing protein aggregates through phagocytosis. However, excessive uptake of these aggregates can compromise their phagocytic capacity, leading to neuroinflammatory reactions and subsequent neuronal degeneration (Gao et al., 2023). Furthermore, the infiltration of peripheral immune cells such as T lymphocytes and B lymphocytes can drive the polarization of microglia toward pro-inflammatory states, thereby accelerating disease progression (Nagatsu et al., 2000). Notably, single-cell sequencing analyses have revealed a strong correlation between the depletion of B cells and the onset and progression of AD. In animal models of AD, the early removal of B cells remarkably exacerbates cognitive impairment (Xiong et al., 2021). Research has demonstrated that CD4^+^ T cells can infiltrate the central nervous system, subsequently triggering microglial activation and neuronal degeneration. This process plays a crucial role in the pathogenesis of numerous neurodegenerative disorders (González and Pacheco, 2014; DeMaio et al., 2022; Su et al., 2023). The constant interplay and dynamic crosstalk between microglia and peripheral immune cells ultimately influence the onset and progression of neurodegenerative disorders.

Dysregulation of m^6^A modification has emerged as a critical factor in the pathophysiology of neurodegenerative diseases, particularly due to its ability to affect the activation and function of glial cells within the neuroimmune microenvironment. The reduced expression of METTL3 renders microglia and astrocytes more active, leading to a secondary immune response condition typically observed after neuronal damage. Analysis of brain tissue from patients with AD showed a remarkable decrease in neuronal m^6^A levels, while m^6^A modification in glial cells was abnormally elevated. This dysregulation was most evident in hippocampal astrocytes, suggesting a strong association between aberrant m^6^A modification and glial cell activation states. At the molecular level, METTL3-mediated m^6^A reduction in neurons triggers adverse changes such as oxidative stress, cell cycle disorders, and apoptosis. The damage signals released by these pathological changes activate surrounding immune cells and create a pro-inflammatory microenvironment. The abnormal m^6^A modification of glial cells is also directly involved in their activation process. This dysregulated immune microenvironment accelerates the progression of neurodegenerative diseases (Zhao et al., 2021). A further mechanistic study revealed that the m^6^A reader YTHDC2 affects the expression of SLC7A11 in astrocytes, disrupting the balance of glutamic acid and thereby causing damage to the neuroimmune environment (Zhang et al., 2024). Immunoprecipitation-methylated RNA profiling demonstrated remarkable hypermethylation of m^6^A in inflammation-related pathways of microglia, providing compelling evidence of a link between m^6^A modification and neuroinflammation. METTL3 activates the TRAF6/NF-κB pathway, which interferes with the regulation of neuronal gene expression and exacerbates LPS-induced neuroinflammation. METTL3 knockdown exerts anti-inflammatory effects by modulating MyD88 alternative splicing, further highlighting the regulatory role of m^6^A modification (Zhang et al., 2022b). In addition to its effect on neurons, abnormal m^6^A modification also alters the immune activity of glial cells and promotes the progression of neurodegenerative diseases through microenvironment remodeling.

In recent years, the role of PCD in neurodegenerative diseases has attracted widespread attention. Research has found that cell death is closely related to the immune microenvironment in the brain. For instance, microglia that exhibit iron accumulation undergo substantial changes in their transcriptional profiles, and these modified microglial cells can induce neuronal degeneration, ultimately leading to cognitive problems in people (Ryan et al., 2023). In addition, ferroptosis exerts pro-inflammatory effects on microglia and macrophages. The use of drugs to inhibit ferroptosis can increase the expression of Nrf2 and alleviate neuroinflammation, thereby offering a novel therapeutic avenue for intervention (Cui et al., 2021). In the context of PD, T cells can recognize the α-synuclein peptides presented by antigen-presenting cells, thereby promoting an inflammatory response in the nervous system. Besides, B lymphocytes can accelerate the apoptosis of midbrain dopaminergic neurons and trigger microglial activation, which is likely to exacerbate PD.

This worsening of the condition may be linked to complex connection with PCD pathways, including ferroptosis (Zheng et al., 2022). Studies have shown the abnormal distribution of copper in the brains of patients with AD leads to the overactivation of microglia and astrocytes. The dysregulated response of these immune cells to pathogen invasion can aggravate neuroinflammation (Choo et al., 2013; Li et al., 2025b). During the neuroinflammatory process related to AD, activated innate immune cells can trigger PCD, such as pyroptosis and necrosis. This cellular demise subsequently releases substances that exacerbate inflammation, thereby intensifying the neuroinflammatory environment (Rajesh and Kanneganti, 2022). As an important approach for the treatment of neurodegenerative diseases, neural regeneration is finely regulated by the local microenvironment. Microglia are key members of the immune microenvironment, and their functional status remarkably impacts immune activities and neuronal survival. Complete microglial ablation reduces both pro-inflammatory and anti-inflammatory mediators, which can alleviate neuroinflammation but may exacerbate neuronal injury in the early stages of the disease. Interestingly, partial microglial depletion is better tolerated by neurons, possibly due to the interaction among the remaining microglia and astrocytes, which forms compensatory mechanisms to maintain neural stability. After microglia regenerate, they can restore immune homeostasis by increasing the expression of neurotrophic factors and enhancing phagocytic clearance capabilities (Li et al., 2021). Classically activated M1 microglia drive neuroinflammation by secreting IL-1β, exacerbating dopaminergic neuron injury, whereas activated M2 microglia exert neuroprotective effects via anti-inflammatory mediators such as IL-10. Such neuroinflammatory cascades are further amplified by reactive astrocytes, which typically potentiate neural damage rather than confer protection. Notably, the CNS immune microenvironment is subject to peripheral modulation, as T lymphocytes have been shown to influence microglial polarization states. This further adds complexity to the immune microenvironment. The peripheral inflammatory response induced by intraperitoneal injection of lipopolysaccharide can inhibit excessive central inflammation, promote the polarization of microglia to an anti-inflammatory phenotype, and induce T cells to infiltrate the midbrain. This process can inhibit M1 microglial activation and protect dopaminergic neurons. This immune regulation exhibits clear time dependence, showing a remarkable protective effect in the short-term (0 days) and medium-term (30 days) stages, but a diminished or absent effect in the long term (90 days). These findings provide a theoretical basis for promoting neural repair by regulating the immune microenvironment (Yang et al., 2022a).

Neurodegenerative diseases arise from the dysregulation of PCD (ferroptosis, cuproptosis, pyroptosis) and an imbalance within the immune microenvironment. Pathological accumulation of iron and copper accumulation drives microglial overactivation, fueling neuroinflammation. These metal disturbances also impair neural regeneration, which is critically dependent on the immune microenvironment. M^6^A has emerged as a key regulator, linking PCD, neuroregeneration, and immune microenvironment. Based on this analysis, targeted intervention of m^6^A modification may delay the pathological progression of neurodegenerative diseases by reshaping the immune microenvironment, and regulating PCD and neural regeneration.

### Therapeutic potential of targeting ferroptosis, cuproptisis, and pyroptosis in neurodegenerative diseases

Existing research demonstrates that m^6^A modification plays a remarkable role in central nervous system (CNS) injury and is involved in many molecules and pathways of the injury (Tian et al., 2022). For instance, in ischemia-reperfusion models, downregulated FTO expression has been reported to exacerbate neuronal damage and apoptosis (Xu et al., 2020a). In sepsis-associated brain injury, emodin was found to inhibit lipopolysaccharide-induced inflammation and pyroptosis in 1321N1 cells by suppressing METTL3-mediated NLRP3 expression (Wang et al., 2022). In oxygen glucose deprivation/re-oxygenation, reduced expression of Lnc-D63785 has been attributed to elevated METTL3-dependent m^6^A methylation of Lnc-D63785, which intensifies the accumulation of miR-422a and neuronal apoptosis (Xu et al., 2020b). Overall, these studies indicate that in brain disease injury models, there are alterations in m^6^A modification levels, and changes in the expression of key m^6^A regulatory factors can affect neuronal damage by influencing PCD. In terms of neuronal repair and regeneration, alterations in m^6^A modification levels and the expression of related proteins can lead to abnormal nervous system functions. These abnormalities include delayed brain tissue development, axonal regeneration disorders, memory alterations, and disturbances in the renewal and differentiation of neural stem cells (Li et al., 2025d). This further highlights its key role in the development of various neuropsychiatric disorders such as depression, AD, and PD (Lei and Wang, 2022). Given the dual nature of the relationship between PCD and nerve injury, repair and regeneration, where it can promote nerve repair by eliminating damaged cells and maintaining stability of the microenvironment, or it can aggravate injury and inhibit regeneration due to excessive activation (Warner et al., 2023; Klemm et al., 2025). Therefore, regulating PCD has become the key to improving neurological prognosis. Notably, there is an interaction between PCD in cells, which further emphasizes the potential value of m^6^A in CNS diseases. Enhancing our understanding of m^6^A and its regulatory role in PCD will provide deeper insights into the crucial role. Overall, PCD mediated by m^6^A modification has emerged as a novel hotspot in disease research. Modulating diverse forms of PCD through regulating m^6^A modification presents promising targets for clinical diagnosis, treatment, and prognosis. This review compiles drugs that affect ferroptosis, cuproptosis, and pyroptosis by regulating m^6^A modification in various diseases (**Additional Table 3**). Overall, most of these agents act on the methyltransferase family, which may be related to the pivotal role of methyltransferases in m^6^A modification.

**Additional Table 3:** Therapeutic potential of targeting ferroptosis, cuproptosis, and pyroptosis in neurodegenerative diseases

Applicable drug	M^6^A regulator	Molecular mechanism	Type of cell death	Disease	Penetrate the blood- brain barrier	Reference
*Shenqi Qiangjing* Granules	METTL3	Inhibit METTL3-GPX4-ferroptosis axis	Ferroptosis	Asthenozoospermia	No	Hilton et al., 2024
*Yanghe Pingchuan* granules	METTL3	Activate the METTL3/P53/SLC7A11 signaling pathway	Ferroptosis	Bronchial asthma	No	Pan et al., 2025
Eranin	METTL3, FTO	Promote m^6^A modification of ALOX12/P53 mRNA	Ferroptosis	Renal cancer	No	Shen et al., 2023
Platycodin D	METTL16	Inhibits METTL16/m^6^A/NUPR1 axis	Ferroptosis	Prostate cancer	No	Sun et al., 2025
Curdione	METTL4, YTHDF2	Promote the expression of METTL14 and YTHDF2	Ferroptosis	Colorectal cancer	No	Wang et al., 2023b
Edaravone	METTL14	Activate METTL14-mediated Aldh1l1 m^6^A methylation	Ferroptosis	Acute central nervous system injury	Yes	Wu et al., 2025a
Arbutin	FTO	Inhibit ferroptosis by FTO/SLC7A11 axis	Ferroptosis	Non-alcoholic fatty liver disease	Yes	Jiang et al., 2023
Plumbagin	YTHDF1	Inhibit YTHDF1 and down-regulate SLC7A5 m^6^A methylation modification	Ferroptosis	Polycystic ovary syndrome	Yes	Cai et al., 2025
Licochalcone A	IGF2BP3	Inhibit the IGF2BP3/MDM2 cascade	Ferroptosis	Acute myeloid leukemia	Yes	Han et al., 2024
Copper ionophores and SIRT2-specific inhibitor	METTL16	Inhibit METTL16 activity through METTL16-K229 lactylation	Cuproptosis	Gastric tumors	No	Sun et al., 2023
chrysin-7-O-glucuronide	ALKBH5	Inhibit the mutual interaction of LRP6 and ALKBH5	Cuproptosis	Myocardial infarction	No	Liu et al., 2025
*Qiju Dihuang* Pill	No study	Inhibit m^6^A/SLC31A1-mediated cuproptosis	Cuproptosis	Diabetic cataract	No	Yang et al., 2024
Berberine	METTL3	Inhibit METTL3 and regulate the ASC/caspase-1/gasdermin D axis	Pyroptosis	Acute kidney injury	Yes	Li et al., 2025a
Wogonin	METTL14	Inhibit METTL14 and m^6^A modification of NLRP3	Pyroptosis	Intracerebral hemorrhage	Yes	Li et al., 2024b
Dexmedetomidine	ALKBH5	Promote mitophagy and inhibit pyroptosis by the ALKBH5/FUNDC1 axis	Pyroptosis	Epidural-related maternal fever	Yes	Xiao et al., 2024
Betaine	YTHDF2	Inhibit NLRP3-mediated microglial pyroptosis through an m^6^A-YTHDF2-dependent pathway	Pyroptosis	Cognitive impairment induced by homocysteine	Yes	Yang et al., 2024b
*Bushen Huoxue* decotion- containing serum	No study	Inhibit STAT3 and downregulate m^6^A modification in chondrocytes	Pyroptosis	Facet joint osteoarthritis	No	Zhou et al., 2024a

Potential drugs that affect ferroptosis, cuproptosis, and pyroptosis by regulating m^6^A modification in various diseases provide a valuable reference for the development of drugs in neurodegenerative disease models. Aldh1l1: Aldehyde dehydrogenase 1 family member l1; ALKBH5: alkB homolog 5; FUNDC1: FUN14 domain containing 1; FTO: fat mass and obesity- associated protein; GPX4: glutathione peroxidase 4; IGF2BP3: insulin-like growth factor 2 mRNA binding protein 3; LRP6: low-density lipoprotein receptor-related protein 6; METTL3: methyltransferase-like protein 3; METTL14: methyltransferase-like protein 14; METTL16: methyltransferase-like protein 16; m^6^A: N^6^-methyladenosine; NLRP3: NLR family pyrin structural domain 3; SLC7A11: solute carrier family 7 member 11; STAT3: signal transducer and activator of transcription 3; YTHDF: YTH domain-containing family.

AD is one of the most common neurodegenerative diseases, characterized by a complex pathophysiology. Despite ongoing research into treatment strategies, an effective cure remains elusive. Delivering pharmaceuticals to the CNS is a complex process and poses a major challenge in formulating therapeutic and preventive strategies (Passeri et al., 2022). Notably, as shown in **Additional Table 3**, there are relatively few disease models for neurodegenerative diseases, and the effectiveness of these drugs in treating CNS diseases depends on their ability to cross the blood–brain barrier. Among the agents listed, Edaravone, Arbutin, Plumbagin, Licochalcone A, Berberine, Wogonin, and Betaine can cross the blood-brain barrier and have applications in CNS diseases. Edaravone can reportedly inhibit ferroptosis in acute CNS injury and exert neuroprotective effects by promoting METTL14-mediated m^6^A methylation of Aldh1l1, suggesting it may delay the progression of AD (Wu et al., 2025a). Although Edaravone has a weak ability to cross the blood-brain barrier and a short half-life, its neuroprotective effect is limited. However, in ischemic stroke models, using microgels to deliver Edaravone can successfully facilitate its crossing of the blood-brain barrier. Compared with free Edaravone, the neuroprotective effect of MG-3-Eda is superior. This drug delivery approach enables Edaravone to target ischemic regions, exerting considerable local antioxidant and anti-inflammatory actions (Lin et al., 2022). Arbutin can also penetrate the blood-brain barrier and is involved in important physiological pathways related to protein misfolding, synaptic function, and neuronal survival processes associated with the development of neurodegenerative diseases. Consequently, it holds therapeutic potential for reducing the risk of neurodegenerative diseases such as Huntington’s disease, amyotrophic lateral sclerosis, PD, and AD (Sharma et al., 2024). Additionally, since Arbutin can inhibit ferroptosis in a fatty liver model by acting on the FTO/SLC7A11 pathway, its role in ferroptosis in AD warrants further investigation (Jiang et al., 2023). Plumbagin exhibits a strong ability to penetrate the blood–brain barrier and can inhibit the growth of gliomas *in vitro* and *in vivo* by targeting NQO1/GPX4-mediated ferroptosis. It can also suppress ferroptosis by inhibiting YTHDF1 and downregulating the m^6^A modification of SLC7A5, suggesting its potential as a novel anti-AD candidate drug (Cai et al., 2025). Licochalcone A exerts regulatory effects on cognition through multiple mechanisms. It enhances cognitive activity via the BDNF-TrkB pathway, reduces neuroinflammation in microglia, inhibits key enzymes involved in tau phosphorylation, and acts as an acetylcholinesterase inhibitor to reduce amyloid plaques. Furthermore, it exhibits multi-target effects, such as antioxidant activity, by activating Nrf2 (Olloquequi et al., 2023). In mouse models, Berberine can reduce brain iron content and prevent rsl3-induced ferroptosis by activating the Nrf2 signaling pathway (Li et al., 2023a). However, there is currently a lack of clinical data to support this view. In neurological diseases, Wogonin treatment has been shown to improve traumatic brain injury in mice, reduce brain edema, and alleviate TLR4/NF-κB-mediated inflammatory responses (Chen et al., 2012). Dexmedetomidine yields neuroprotective effects, including protection of the blood-brain barrier, reduction of neuronal death, maintenance of hemodynamic stability, and alleviation of cognitive dysfunction (Tao et al., 2024). Moreover, it can exert various neuroprotective effects, including anti-inflammatory and antioxidant stress effects, regulation of autophagy, and reduction of apoptosis in brain diseases. However, its role in ferroptosis, cuproptosis, and pyroptosis in AD remains to be clarified. Betaine has been reported to cross the blood–brain barrier and participate in regulating hippocampal neurophysiology and neuroprotection, although its regulatory role in disease models remains insufficient (Knight et al., 2017). Furthermore, research indicates that nano-liposomes and exosomes, as intelligent drug delivery systems, can penetrate the blood–brain barrier and target brain tissue (Passeri et al., 2022). For other drugs that are unable to cross the blood-brain barrier but can act on ferroptosis, cuproptosis, and pyroptosis through m^6^A, exploring auxiliary methods to facilitate their role in the disease process of AD presents new challenges for the research and development of AD drugs and clinical treatment.

**Additional Table 3** provides a summary of application drugs targeting the m^6^A-programmed cell death axis. Currently, most m^6^A regulatory drugs have been primarily studied in non-neurological disease models, and their mechanisms and effects in neurodegenerative diseases remain to be verified. Moreover, the clinical application of some promising drugs is limited due to their poor permeability across the blood–brain barrier. This challenge has prompted more researchers to focus on developing drug delivery systems. While the effects of these drugs in AD have not yet been confirmed, their regulatory effects on ferroptosis, cuproptosis, and pyroptosis by targeting m^6^A regulatory factors have been demonstrated. By adopting optimized drug delivery and targeting strategies, such as precisely inhibiting excessive PCD or moderately inducing PCD, these drugs may offer novel therapeutic targets for the clinical treatment of neurodegenerative diseases.

## Death–Regeneration Balance in Neurodegenerative Diseases

### Mechanisms of neural regeneration in neurodegenerative diseases

Mature neurons in the central nervous system lack the ability to regenerate after injury, a hallmark of many neurological disorders. Nonetheless, recent studies have demonstrated that certain regions of the adult brain, particularly the dentate gyrus of the hippocampus, retain a limited potential for neurogenesis (Duan et al., 2008; Dumitru et al., 2025). Notably, transplantation of exogenous cells or differentiation of induced pluripotent stem cells into neurons can enhance hippocampal neurogenesis (Wu et al., 2021). Neural stem cells promote neurogenesis, maintain synaptic connections, and support synapse formation by secreting neurotrophic factors such as BDNF and nerve growth factor, thereby improving cognitive function (Rauti et al., 2020). Modulating the phenotype of microglia can further promote neural regeneration and synaptic remodeling (Man et al., 2019). However, in AD, the capacity for neural regeneration is markedly impaired, which is believed to be a key pathological basis for the progressive worsening of neurodegenerative lesions.

The impaired neuroregenerative capacity in neurodegenerative diseases arises from multifaceted pathological mechanisms, with mitochondrial metabolic dysfunction emerging as a central contributor. In neurons, mitochondrial impairment manifests as disrupted energy homeostasis, characterized by inefficient oxidative phosphorylation and ATP depletion. These bioenergetic deficiencies critically undermine synaptic plasticity and memory-related neuronal functions. Compounding this dysfunction, structural abnormalities in mitochondria further reduce their capacity to meet cellular energy demands. Dysfunctional mitochondria also generate excessive ROS, creating oxidative stress that exacerbates neuronal degeneration. The neurotrophic factor family, particularly nerve growth factor and its homologs, constitutes essential components of neural regeneration. Patients with neurodegenerative diseases consistently exhibit diminished expression of these critical neurotrophic factors, and this deficiency has been associated with early cognitive impairment (Enciu et al., 2011; Tuszynski et al., 2015). Epigenetic regulation has emerged as another pivotal determinant of neuroregenerative capacity in neurodegenerative diseases. Complex epigenetic modifications, including DNA methylation, dynamic histone modifications, and non-coding RNA-mediated regulation, are now understood to control the fate of neural stem cells. For example, promoter hypermethylation or chromatin compaction may silence transcription factors and disrupt signaling cascades that are critical for neuronal repair (Gräff et al., 2012). The neuroregenerative process also faces challenges from the inflammatory microenvironment characteristic of AD, where activated microglia release inflammatory mediators that not only induce neuronal damage but also exacerbate it (Lee and Kim, 2017).

### N^6^-methyladenosine modification and neural regeneration

In recent years, m^6^A modification has emerged as a pivotal epigenetic mechanism in neural regeneration research. Studies have demonstrated its fundamental involvement in multiple aspects of neural repair, particularly through regulating neural stem cell activation, differentiation and axonal regeneration processes (Shafik et al., 2022; Lin et al., 2023). The m^6^A modification serves as a critical molecular switch that determines cellular fate between regeneration and death. The methyltransferase METTL3, as the core catalytic component, exhibits indispensable functions in neural stem cell regeneration. Experiments have shown that the loss of METTL3 leads to a decrease in m^6^A in activated neural stem cells, resulting in impaired proliferation and altered differentiation pathways by promoting glial cell formation and inhibiting neuronal development *in vivo* and *in vitro*. A mechanistic study has also shown that METTL3 depletion results in a marked decrease in enhancer of zeste homolog 2 (Ezh2) protein expression (Chen et al., 2019). Ezh2 overexpression demonstrates rescue of neural developmental deficits, suggesting that m^6^A-dependent control of Ezh2 expression plays a critical role in coordinating neural stem cell proliferation, differentiation, and neuronal maturation (Chen et al., 2019). The regenerative capacity of neurons in the central nervous system is extremely limited, which poses a huge challenge to the development of neural repair therapies. The m^6^A modification plays an important role in the process of neural regeneration. For example, ALKBH5 protein can promote axon regeneration by maintaining the stability of Lpin2 mRNA, and this mechanism is closely related to the regulation of lipid metabolism (Wang et al., 2023a). In the context of peripheral nerve injury, key m^6^A target genes, including *Atf3*, *Creb1* and *Jun*, are closely related to the functional recovery. The loss of *Atf3* considerably weakens the regenerative capacity of dorsal root ganglion neurons, while the upregulation of *Creb1* enhances axon regeneration. Knockdown of *Jun* not only delays axon regeneration, but also disrupts the reconstruction of functional synaptic connections (Zhang et al., 2021). These findings indicate that m^6^A modification not only affects the differentiation of neural stem cells but also plays multiple roles in neuronal development and injury repair, providing important clues for the development of new neural regeneration strategies.

### Relationship between programmed cell death and neural regeneration

PCD is not merely a passive consequence of nerve injury. Emerging evidence indicates that, in some cases, PCD can promote repair and functional recovery (Guo et al., 2024; Klemm et al., 2025). Following sciatic nerve injury, an increase in thrombin levels activates the ferroptosis pathway, leading to Schwann cell death and blocked myelin regeneration. Studies have found that thrombin reduces the phosphorylation of yes-associated protein (YAP) protein in the Hippo signaling cascade through the protease-activated receptor 1 (PAR1) receptor, promoting YAP nuclear translocation and ACSL4 gene expression, which ultimately triggers lipid peroxidation and cell death. Inhibition of PAR1 effectively downregulates the expression of YAP and ACSL4, thereby alleviating ferroptosis and creating a favorable microenvironment for axon regeneration (Wu et al., 2025b). After peripheral nerve injury, the accumulation of a large amount of ROS accelerates ferroptosis and neuronal loss, forming a major obstacle to nerve regeneration. This review revealed that the use of iron chelators, ferroptosis inhibitors, or HYD could protect cells, promote axon regeneration, and even improve motor function (Li et al., 2024c). In the central nervous system, neuronal ferroptosis caused by intracerebral hemorrhage can remarkably interfere with neuroregeneration. Platelet factor 4 can enhance the expression of SLC7A11 through the CXCR3/AKT1/SLC7A11 pathway, reducing ferroptosis, mitigating inflammation, and aiding in nerve regeneration (Hu et al., 2025). This demonstrates a clear correlation between the degree of ferroptosis and nerve regeneration, establishing a concentration-dependent relationship. Different degrees of ferroptosis elicit varied responses in neurons. For example, GPX4 gene deletion or exposure to low concentrations of ferroptosis inducers can promote axon fusion, while high concentrations of inducers can lead to structural collapse (Ko et al., 2025). Lipid peroxidation levels are crucial to the injury response pattern. Phosphatidylserine externalization helps axons reconnect. Moderate oxidative stress triggers phosphatidylserine externalization, a key step in achieving PSR-1 and EFF-1-mediated fusion. The ferroptosis inducer ML162 has been shown to leverage this mechanism to improve functional recovery after nerve injury (Ko et al., 2025). Excessive ferroptosis can disrupt Schwann cell myelination and neuronal viability, thereby impairing regeneration (Yoon et al., 2017). However, moderate regulation can stimulate repair through axonal fusion. Therefore, precise intervention in key molecules such as YAP, ACSL4, SLC7A11, Nrf2, and Fpn may represent a new direction for future neural regeneration therapy.

Copper ions play a complex role in the process of nerve regeneration, exhibiting concentration-dependent effects on neuronal function. Normal physiological levels of copper are now understood to be beneficial to neurons. Copper not only enhances the activity of neurotrophic factors but also directly promotes axon growth and neuronal survival. Copper ions aid neurite extension by interacting with PrPC proteins, which are essential for maintaining normal neuronal function. In an amyotrophic lateral sclerosis model, appropriate copper supplementation was found to delay disease progression (Nicoletti et al., 2022), further substantiating the importance of copper homeostasis. Conversely, excessive copper yields the opposite effect. Elevated copper concentrations not only interfere with receptor binding, leading to neurotoxicity and regenerative disorders, but also hinder nerve regeneration by disrupting the CREB/BDNF pathway (Zhang et al., 2023b; Xing et al., 2024b). More importantly, excessive copper can induce cuproptosis, which is highly detrimental to nerve regeneration. This highlights the need for precise copper balance in the nervous system. Therefore, a deeper understanding of the relationship between cuproptosis and nerve regeneration may provide important clues for the development of new nerve repair therapies. Future research should focus on how to precisely regulate copper levels to harness its pro-regenerative effects while avoiding toxic outcomes.

Copper ions play a complex role in the process of nerve regeneration, exhibiting concentration-dependent effects on neuronal function. Normal physiological levels of copper are now understood to be beneficial to neurons. Copper not only enhances the activity of neurotrophic factors but also directly promotes axon growth and neuronal survival. Copper ions aid neurite extension by interacting with PrPC proteins, which are essential for maintaining normal neuronal function. In an amyotrophic lateral sclerosis model, appropriate copper supplementation was found to delay disease progression (Nicoletti et al., 2022), further substantiating the importance of copper homeostasis. Conversely, excessive copper yields the opposite effect. Elevated copper concentrations not only interfere with receptor binding, leading to neurotoxicity and regenerative disorders but also hinder nerve regeneration by disrupting the CREB/BDNF pathway (Zhang et al., 2023b; Xing et al., 2024b). More importantly, excessive copper can induce cuproptosis, which is highly detrimental to nerve regeneration. This highlights the need for precise copper balance in the nervous system. Therefore, a deeper understanding of the relationship between cuproptosis and nerve regeneration may provide important clues for the development of new nerve repair therapies. Future research should focus on how to precisely regulate copper levels to harness its pro-regenerative effects while avoiding toxic outcomes.

### Impact of N^6^-methyladenosine-regulated programmed cell death on neural regeneration

**Figure 6:**
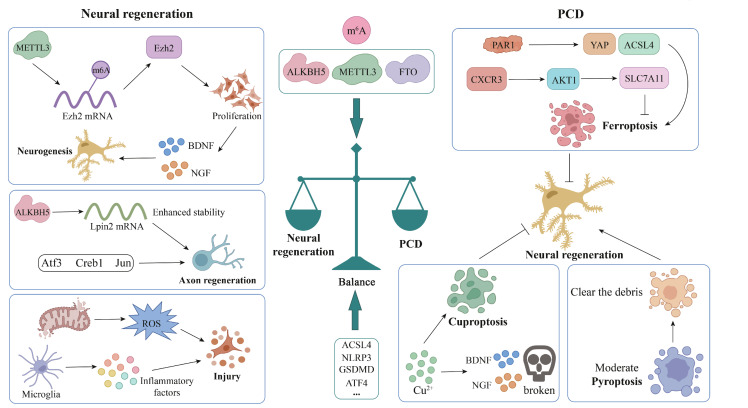
M^6^A influences neural regeneration by regulating programmed cell death and participating in the dynamic balance between cell death and regeneration. M^6^A regulates neural regeneration by controlling neural stem cell differentiation, axon regeneration, and cellular injury responses. Moderate programmed cell death promotes nerve regeneration, while uncontrolled cell death inhibits it. M^6^A maintains the balance between death and regeneration by regulating key targets such as ACSL4, NLRP3, GSDMD, and ATF3. ACSL4: Acyl-CoA synthetase long-chain family member 4; AKT1: AKT serine/threonine kinase 1; ALKBH5: alkB homolog 5; BDNF: brain-derived neurotrophic factor; CXCR3: C–X–C motif chemokine receptor 3; Ezh2: enhancer of zeste homolog 2; FTO: fat mass and obesity-associated protein; METTL3: methyltransferase like 3; m^6^A: N^6^-methyladenosine; NGF: nerve growth factor; PAR1: protease-activated receptor 1; PCD: programmed cell death; SLC7A11: solute carrier family 7 member 11; YAP: yes-associated protein.

The m^6^A modification plays a key role in regulating the “death-regeneration balance” by influencing the cell’s response to injury and various stages of nerve regeneration, ultimately determining cell fate. Studies have shown that m^6^A modification can regulate nerve cell responses to injury by affecting death-related factors such as ACSL4, NLRP3, GSDMD, and ATF4 mRNA (Feng et al., 2024a; Yang et al., 2024b). Importantly, m^6^A modification can regulate neuronal sensitivity to lipid peroxidation and ferroptosis by modulating ACSL4. The expression of SLC7A11 can promote nerve regeneration and treatment with the ferroptosis inducer erastin can reduce the proliferation rate of neural stem cell by up to 50% (Xing et al., 2024a). It is worth noting that SLC7A11 is also a target gene regulated by m^6^A modification, which governs ferroptosis by regulating the stability and expression level of SLC7A11 mRNA, thereby playing a key role in regulating nerve regeneration. During axon regeneration, insufficient mitochondrial energy triggers compensatory mechanisms such as increased mitochondrial transport and upregulation of local glycolysis (Masin et al., 2024). However, an abnormal accumulation of copper ions disrupts mitochondrial function and inhibits regeneration. Cuproptosis-related genes such as FDX1, PDHA1, and PDHB have been found to be closely related to glycolysis (Qin et al., 2024). As a mitochondrial protein, FDX1 participates in cuproptosis by regulating Fe-S cluster binding and LIAS-mediated protein acylation, affecting the interaction between copper ions and acylated proteins and abnormal oligomerization (Qin et al., 2024). As mentioned above, METTL16 affects cuproptosis by modifying FDX1 mRNA through m^6^A. During the inflammatory response, m^6^A regulates the expression of pyroptosis signaling molecules such as NLRP3 and GSDMD to prevent excessive inflammatory damage and protect nerve function. After sciatic nerve injury, the mRNA expression of m^6^A-related methyltransferases and demethylases was significantly increased compared with the control tissue. Among them, FTO and METTL14 expression were considerably upregulated, indicating that these enzymes promote repair by selectively regulating regeneration-related genes and the inflammatory microenvironment (Zhang et al., 2021). Interestingly, m^6^A can inhibit overactivated pyroptosis and promote nerve regeneration (Li et al., 2025d). Current evidence suggests that m^6^A affects nerve regeneration through cell PCD and regulates the dynamic balance between cell death and regeneration (**Figure 6**). This multi-level regulatory mechanism makes m^6^A a potential therapeutic target to optimize the regenerative microenvironment by coordinating pathways such as cuproptosis, glycolysis, and pyroptosis.

## Current Status of Clinical Translation and Application

Based on current research on m^6^A modification, PCD, and neurodegenerative diseases, the development of new therapeutic targets and drugs has become a major research hotspot. We are examining the ClinicalTrials.gov (https://clinicaltrials.gov/), the National Institutes of Health (NIH), the Chinese Clinical Trial Registry (ChiCTR), and the Food and Drug Administration (FDA) to summarize new therapeutic targets or drugs for neurodegenerative diseases related to ferroptosis, pyroptosis, and m^6^A modification. This review highlights future research directions and potential in the field. Notably, no trials related to this topic were found in the ClinicalTrials.gov or ChiCTR, and no new drugs targeting this pathway have been approved in the FDA database. However, the NIH database contains numerous clinical research and drug development projects related to the topic of this article. We have listed and summarized the main aspects of these projects, including project name, funding agency, research objectives, main results, and research status (**Additional Table 4**). The role of ferroptosis in neurodegenerative diseases involves the regulation of lipid metabolism, oxidative stress, and iron homeostasis. Previous studies have identified the key molecular mechanisms of ferroptosis by developing small molecule inhibitors, using high-throughput screening and proteomics methods, and exploring its potential as a therapeutic target. Some of these studies have been completed and have entered the translational stage (Yang et al., 2019; Protchenko et al., 2021; Cardona et al., 2024). Clinical research on cuproptosis in neurodegenerative diseases is currently limited but holds great potential. Research on pyroptosis focuses on the activation mechanisms of NLRP3 and NLRP6 inflammasomes and their roles in neuroinflammation and infectious diseases. This research has provided new ideas for developing anti-inflammatory and neuroprotective strategies by studying the regulation of pyroptosis by HIV Tat and morphine, the induction mechanism of Dipeptidyl Peptidases 8/9 (DPP8/9) inhibitors, and the inhibitory effect of neurosteroids on GSDMD.

**Additional Table 4:** Clinical research on m^6^A modification and programmed cell death as targets in neurodegenerative diseases

Project name	Funding agency	Research objective	Main result	Research status	Study in the similar fields
Spatial metabolomics with subcellular resolution to identify therapeutic targets	National Institute of General Medical Sciences	Investigate lipid metabolism-driven cell death through the modulation of ferroptosis in cell culture and *in vivo* models of drug-resistant cancer.	Providing fundamental insights into the molecular chemistry of ferroptosis and its role in disease pathology by developing multi-omics approaches with single-cell resolution.	Ongoing	Allerton et al., 2024
Toxicology in the 21^st^ century program (Tox21)- genomic toxicology	National Center for Advancing Translational Sciences	Through multi-institutional collaboration, the scholars used human cell culture models and high- throughput screening technologies to elucidate the toxicological mechanisms of environmental toxicants and drugs on the cardiovascular and nervous systems, and to evaluate and screen potentially toxic compounds.	*Tong Zhibin* developed and validated a high- throughput 3D LUHMES cell screening method. Using this method, he identified 64 compounds that may trigger ferroptosis, leading to neuronal death, by disrupting metal distribution and inducing oxidative stress. These compounds may be potential causative factors for Parkinson's disease and other neurodegenerative diseases.	Completed	Tong et al., 2022; Tong et al., 2024
Expanding the facets of multi-functional antioxidant molecules through computational design and biological tools	National Institute of General Medical Sciences	Develop multi-mechanistic antioxidant small molecules to prevent oxidative stress and ferroptosis in neurodegenerative diseases, and screen for drug candidates with good metabolism and blood-brain barrier permeability.	Screening of small molecules that effectively activate Nrf2 and resist ferroptosis, thereby protecting cells from oxidative stress damage in cell models, with favorable drug properties to support subsequent translational research.	Ongoing	Johnston et al., 2019
Development of MESH1 inhibitors to treat ferroptosis-associated neurodegeneration	National Institute on Aging	Develop and optimize small molecule inhibitors targeting MESH1 to block ferroptosis to protect neurons in Alzheimer's disease and Alzheimer's- related dementia models, and promote the clinical translation of ferroptosis mechanisms in neurodegenerative diseases.	The scholars identified and validated candidate small molecules with the potential to inhibit MESH1 activity, including a quinazolinone and a triazino-indole. These inhibitors effectively suppressed ferroptosis in both cell models and brain tissue slice models, demonstrating promising therapeutic potential.	Completed	Yang et al., 2019
Characterization and modulation of ferroptosis by chemical proteomics	National Cancer Institute	The scholars used innovative chemical probes and proteomic methods to analyze the mechanism of cytotoxicity of oxidized phospholipids and their metabolites during ferroptosis. They identified key phospholipases and their targets that regulate ferroptosis and mapped the reactivity of cysteine related to ferroptosis, providing a biological basis for developing therapeutic strategies to promote or inhibit this process.	The scholars established a method for screening serine phospholipase inhibitors and their targets, revealing their regulation of oxidized phospholipids in cancer cells. They quantified cysteine reactivity in ferroptosis, identified key protein sites for lipid- derived electrophilic reagents, and determined significant protein modifications and functional changes.	Completed	Kathman et al., 2020
Design and selection of novel metalloenzymes for biocatalysis, bioimaging, and genetic engineering	National Institute of General Medical Sciences	Design and screen two types of metalloenzymes: metalloproteinases and metalloDNAzymes. Gain a deeper understanding of their structure and function, and develop application platforms for biocatalysis, bioimaging, and gene editing, with a particular focus on the role of metal ions in cells and their association with neurodegenerative diseases such as ferroptosis.	The scholars constructed a multimetallic protein model to analyze the relationship between metalloenzyme structure and activity. They screened for highly selective DNA enzymes to enable simultaneous imaging of Fe^2+^ and Fe^3+^ in living cells. They developed a DNA enzyme gene editing tool to enhance the efficiency of gene cloning and editing.	Ongoing	Wu et al., 2023; Banik et al., 2024
Contributions of iron regulatory proteins and ferroptosis to neurodegeneration	National Institute on Aging	To investigate the role of IRP1 and IRP2 in regulating neuronal ferroptosis and the impact of IRP-mediated iron homeostasis imbalance on tau- related neurodegenerative diseases.	Increased IRP activity promoted iron accumulation and ferroptosis, while tau protein pathology aggravated iron efflux disorders and neurodegeneration; zebrafish models showed that inhibiting ferroptosis can slow down IRP- dependent tau-mediated neurodegeneration.	Completed	Cardona et al., 2024
Lipid droplets and the regulation of ferroptosis	National Institute of General Medical Sciences	To study the subcellular localization of the two splicing isoenzymes of ACSL4 and their regulatory mechanism on ferroptosis sensitivity, reveal how the sequestration of lipid peroxides between the cell membrane and lipid droplets affects the ferroptosis process, and evaluate the potential of ACSL4 as a therapeutic target for anti-cancer and neurodegenerative diseases.	Endogenous ACSL4-deficient cell lines were constructed and isoenzymes with different subcellular localizations were expressed. It was found that lipid droplet-localized isoenzymes reduced ferroptosis sensitivity by sequestering lipid peroxides; lipid analysis supported the key regulatory role of ACSL4 in ferroptosis.	Completed	Morris et al., 2022
Elucidating the relationship between lipid droplets, lipid metabolism, and lipotoxicity	National Institute of General Medical Sciences	Through genome-wide CRISPR screening, the scholars identified and analyzed the mechanism of action of lipid droplet oxidoreductase AIFM2 in cancer cell resistance to ferroptosis, studied the relationship between lipid droplet and fatty acid metabolism and ferroptosis, and identified new factors that protect cancer cells from ferroptosis.	AIFM2 is a key factor in cancer cell resistance to ferroptosis, and its loss increases sensitivity to ferroptosis. The study uncovered a novel mechanism by which AIFM2 prevents lipid damage, highlighting the role of lipid droplets and fatty acid metabolism in the regulation of ferroptosis.	Ongoing	Roberts et al., 2023
Identification of human genes of iron homeostasis	National Institute of Diabetes and Digestive and Kidney Diseases	To study the role of PCBP1, PCBP2, and BOLA2 in ferroptosis, and review the imbalance of iron, thiol, and phospholipid metabolism that causes neuronal ferroptosis and its treatment strategies.	PCBP1 deficiency in hepatocytes disrupts iron homeostasis and leads to lipid peroxidation, while antioxidant therapy effectively improves liver disease. PCBP1 is essential for maintaining the activity of effector T cells; its deficiency results in an increase in regulatory T cells and impairs anti-cancer immunity. Additionally, PCBP2 collaborates with PCBP1 to stabilize cytokine mRNA and is regulated by zinc ions. Reduced copy number of BOLA2 is associated with iron deficiency anemia, and animal studies support its role in maintaining iron homeostasis. Ferroptosis depends on lipid peroxidation, iron catalysis, and thiol regulation, highlighting a new target for neurodegenerative diseases.	Completed	Protchenko et al., 2021
The Hippo signaling pathway as a target of intervention for Alzheimer's disease	National Institute on Aging	To fill a significant knowledge gap regarding the significance of Hippo signaling overactivation in AD and provide the first proof-of-concept evidence for inhibiting Hippo signaling as a new therapeutic strategy for AD.	Overactivation of the Hippo signaling pathway was found in AD models. Genetic knockout of Lats1 and Lats2 inhibited Hippo signaling, significantly improving cognitive function and reducing neurodegenerative damage in AD mice. Furthermore, inhibiting Hippo signaling enhanced neuronal resistance to ferroptosis, providing new insights into AD treatment.	Ongoing	Mueller et al., 2018
Investigating the role of apolipoprotein E in brain ferroptosis sensitivity	National Institute on Aging	To study how different apoE isoforms and their interactions with lipids, especially HDL, affect the sensitivity of central nervous system cells to ferroptosis; verify the regulatory effects of apoE isoforms and lipid components on ferroptosis through cell and organ slice models, and identify the key lipid types that confer ferroptosis resistance.	Astrocytes and microglia are susceptible to ferroptosis when antioxidants are suppressed. Astrocytes expressing apoE4 are more susceptible to ferroptosis than those expressing apoE3/apoE2. During ferroptosis, cells accumulate damaged lipids and triglycerides to protect polyunsaturated fatty acids. Cells lacking apoE4 and apoE are metabolically stressed, their protective abilities weakened, and their susceptibility to ferroptosis is increased.	Completed	Richards et al., 2025
Development of ferroptosis inhibitors for Huntington disease	National Institute of Neurological Disorders and Stroke	A panel of optimized compounds was developed to investigate the role of lipid peroxidation in a Huntington's disease mouse model *in vivo*. These compounds will be used to investigate the roles of lipid peroxidation and ferroptosis in various degenerative diseases.	This program has developed fourth- and fifth- generation ferroptosis inhibitors. Fifth-generation ferroptosis inhibitors are well tolerated in both wild-type and symptomatic R6/2 Huntington's disease mice and are suitable for evaluating the role of ferroptosis in animal models of degenerative diseases and other ferroptosis- related brain disorders.	Completed	Upadhyayula et al., 2023
Identification of essential sites of lipid peroxidation in ferroptosis using Raman spectroscopy imaging	National Institute on Aging	To determine the subcellular localization of ferroptosis inhibitors to identify drug targets for inhibiting neurodegenerative oxidative cell death. To identify essential membranes for inhibiting and inducing neuronal ferroptosis by targeting lipid peroxidation to modulate sensitivity to ferroptosis.	The cellular localization sites of ferroptosis inhibitors have been preliminarily determined, revealing the key subcellular structures where lipid peroxidation occurs. By targeting and regulating these sites to influence ferroptosis sensitivity, we have deepened our understanding of the mechanism of ferroptosis and provided potential targets and technical support for the development of disease-modifying drugs.	Completed	von Krusenstiern et al., 2023
Elucidating the functional mechanism of NLRP3 inflammasome activation	National Institute of Allergy and Infectious Diseases	To elucidate functional mechanisms of NLRP3 inflammasome activation by studying conformational transitions and intracellular trafficking.	Guiding the development of NLRP3-targeted therapies	Ongoing	Fu et al., 2024
HIV Tat and morphine- mediated pyroptosis activates astrocytes: Role of NLRP6 inflammasome in HAND	National Institute on Drug Abuse	To investigate the epigenetic mechanism of microRNA-152-regulated NLRP6 inflammasome activation in astrocytes under the combined effects of HIV Tat and morphine, and to verify its *in vivo* effects.	Preliminary sequencing data indicate that increased DNA hypomethylation of the NLRP6 promoter is accompanied by upregulated NLRP6 expression in the frontal cortex of SIV-infected rhesus macaques. Exposure of human and mouse primary astrocytes to HIV Tat and morphine decreases microRNA-152 expression, increases NLRP6 expression, and enhances cell activation, promoting the production of pro-inflammatory cytokines, such as IL-1β and IL-18 through pyroptosis.	Completed	Kannan et al., 2023
Characterizing the mechanism of DPP8/9 inhibitor-induced pyroptosis	National Institute of Allergy and Infectious Diseases	To determine how the inhibition of DPP8/9 induces pyroptosis in monocytes and macrophages.	Elucidating the original functions of these enigmatic inflammasomes has revealed a previously unknown link between intracellular redox state and protein stability.	Ongoing	Hollingsworth et al., 2021
Neurosteroid inhibition of pyroptotic lysis	National Institute of Allergy and Infectious Diseases	To explore the inhibitory effects of pregnenolone sulfate and other steroids on cell pyroptosis and its molecular mechanism, verify their effects on gasdermin D pore formation, lysis, membrane localization and oligomerization, analyze the relationship between steroid structure and function, promote the development of highly effective and specific steroid molecules, and provide new strategies for preventing cell pyroptosis and lysis.	Pregnenolone sulfate blocks pyroptotic cell lysis without affecting inflammasome and caspase-1 activation, suggesting that it may act by regulating pore formation or membrane repair mechanisms. The inhibitory effect of steroids on pyroptotic cell lysis is related to their molecular structure, supporting further screening and optimization of steroid drug development.	Completed	Huston et al., 2023
Control of RNA methylation by growth signals through the mTORC1 pathway	National Institute of General Medical Sciences	To decipher the molecular mechanisms by which mTORC1 controls RNA methylation in normal and pathological settings	MTORC1 is a target for treating neurodegenerative diseases.	Ongoing	Villa et al., 2021
Childhood-onset hypomyelinating leukodystrophy and the multi-tRNA synthetase complex	National Institute of Neurological Disorders and Stroke	The effect of insufficient EPRS1 expression on myelination of oligodendrocytes	The EPRS1P1482T mutation affects EPRS1 mRNA nuclear export and translation efficiency through m6A methylation.	Ongoing	Khan and Fox, 2023
m^6^A RNA modification as a regulator of R-loop homeostasis and gene expression in aging and neurodegeneration	National Institute on Aging	To reveal the role of the m^6^A-R-loop axis in brain aging and neurodegenerative diseases	Restoring m^6^A homeostasis or enhancing R-loop expression can delay neurodegeneration.	Completed	Hall, 2023
Effects of mRNA m^6^A methylation and its YTHDF reader proteins on human AD mRNA homeostasis	National Institute on Aging	To explore the regulatory role of m^6^A methylation and its reading protein YTHDF in AD.	YTHDF regulates mRNA stability by recognizing the m^6^A modification.	Completed	Li et al., 2023b
Novel epitranscriptomic mechanisms in metal neurotoxicity	National Institute of Environmental Health Sciences	To explore the anti-inflammatory function of YTHDF2 in the neurotoxicity of Mn.	YTHDF2 inhibits the release of pro-inflammatory cytokines by targeting the SEKl/JNK/c-Jun signaling axis.	Ongoing	Miller et al., 2025
METTL3-mediated regulation of motor neuron function	National Institute on Aging	To explore the influence of m^6^A modification on the expression of TDP-43.	Loss of m^6^A leads to abnormally elevated TDP-43 protein levels.	Ongoing	Dermentzaki et al., 2024
The *in vivo* role of m^6^A RNA modification in cellular stress, aging, and neurological disease	National Institute on Aging	To explore the function of the m^6^A reading protein CG6422 in neurodegeneration.	CG6422 knockout reduces TDP-43 protein expression.	Completed	Perlegos et al., 2024
Neuronal silencing of ATXN3 using peripherally administered antibody/ASO conjugates that penetrate the blood-brain barrier	National Institute of Neurological Disorders and Stroke	To develop a novel bispecific antibody-ASO conjugate (bAb-ASO) delivery system.	After intravenous injection of bAb-ASO, ATXN3 protein levels decreased significantly, delaying motor dysfunction.	Ongoing	Veire et al., 2025

We summarized the research projects funded by NIH, and identified new targets and intervention strategies from several aspects including project names, funding agencies, research objectives, main results, and research status. ACSL4: Acyl-CoA synthetase long-chain family member 4; AD: Alzheimer's disease; AIFM2: apoptosis-inducing factor mitochondrial 2; ASO: antisense oligonucleotide; ATXN3: ataxin 3; BOLA2: bolA family member 2; CBP1: calcium binding protein 1; CRISPRP: clustered regularly interspaced short palindromic repeat; DPP8/9: dipeptidyl peptidases 8/9; EPRS1: glutamyl-prolyl-tRNA synthetase 1; HDL: high-density lipoprotein; HIV Tat: human immunodeficiency virus trans-activator of transcription; IL-18: interleukin-18; IL-1β: interleukin-1 beta; IRP: iron regulatory protein; m^6^A: N^6^-methyladenosine; MESH1: metazoan SpoT homolog-1; METTL3: methyltransferase-like 3; mTORC1: mechanistic target of rapamycin complex 1; NLRP3: NLR family pyrin domain containing 3; NLRP6: NLR family pyrin domain containing 6; PCBP2: poly (C)-binding protein 2; TDP-43: TAR-DNA binding protein of 43 kDa; Tox21: toxicology in the 21^st^ century program; YTHDF: YTH domain-containing family protein.

Some of these studies have achieved significant results (Hollingsworth et al., 2021; Huston et al., 2023; Kannan et al., 2023; Fu et al., 2024). The regulatory effect of m^6^A modification on neurodegenerative diseases involves the functions of the mTORC1 pathway, R-loop homeostasis, and YTHDF reading proteins. Research has found that m^6^A modification participates in the disease process by influencing mRNA stability and protein expression (such as TDP-43). Some projects have been completed, and their therapeutic potential has been verified, while other studies are still ongoing (Villa et al., 2021; Hall, 2023; Dermentzaki et al., 2024; Miller et al., 2025). These studies have provided new targets and intervention strategies for the treatment of neurodegenerative diseases, demonstrating great potential for clinical application.

## Limitation

Although this review focuses on the role of m^6^A modification in regulating the balance between ferroptosis, cuproptosis, pyroptosis, and neural regeneration in neurodegenerative diseases, it is important to acknowledge that other epigenetic modifications and PCDs, such as DNA methylation, histone modification, m5C modification, disulfidptosis, autophagy, and necroptosis, also play significant roles. In this context, Dnmt3a-mediated DNA methylation is a key factor in motor neuron apoptosis, and its abnormal activation has been associated with neurodegenerative diseases such as amyotrophic lateral sclerosis (Chestnut et al., 2011). While this review aims to explain the specific mechanisms, there exists a complex mutual regulatory relationship between m^6^A modification, PCDs, and neural regeneration. Currently, many molecular mechanisms remain in the exploratory stage. For instance, cuproptosis, a recently discovered form of cell death, still lacks comprehensive research. Although current models suggest potential mechanisms for coordinated PCD regulation in neural tissues, conclusive experimental validation of these pathways has yet to be established.

## Discussion

Metal ions such as iron, copper, and zinc accumulate in the brains of patients with AD. Changes in metal ion homeostasis have been associated with Aβ deposition and tau protein hyperphosphorylation, promoting oxidative stress and intracellular metabolic disorders that lead to various forms of PCD. While this review focuses on ferroptosis, cuproptosis, and pyroptosis, other forms of programmed cell death also play important roles in the occurrence and progression of neurodegenerative diseases. For instance, autophagy and necroptosis remain significant areas of study in this context. Although the association between autophagy and necroptosis has been preliminarily confirmed, their precise roles in neurodegenerative diseases are not yet fully understood. While this review does not comprehensively discuss other modes of programmed cell death, their mechanisms are integral to neurodegenerative diseases and warrant further investigation and summary in the future.

There is complex crosstalk between different PCD pathways that determines cell fate and regulates disease progression. Ferroptosis triggers neuroinflammation through a STING-dependent DNA sensing pathway, activating inflammasomes and initiating pyroptosis (Liu et al., 2023). This indicates that ferroptosis and pyroptosis can mutually promote each other through inflammatory mechanisms. Additionally, copper-dependent GPX4 autophagy also promotes ferroptosis (Xue et al., 2023). Furthermore, Erastin induces ferroptosis to produce ROS, thereby triggering other forms of PCD, such as autophagy (Park and Chung, 2019). These findings suggest that ferroptosis, cuproptosis, and pyroptosis form an interconnected network that influences one another through specific molecular mechanisms and synergistically determines cell fate. Understanding these interactions is essential for developing treatment strategies against neurological diseases.

Epigenetic modifications, including DNA methylation, histone modification, and non-coding RNA modification, induce cell death by regulating the expression of key proteins. Among histone modifications, the monoubiquitination of histone H2B is significantly reduced during the induction of ferroptosis, and its loss enhances the sensitivity of cells to ferroptosis (Wang et al., 2019). Additionally, there are interactions between epigenetic modifications: DNA methylation can influence the location and type of histone modifications, while histone modifications can regulate DNA methylation. The interactions between these modifications are crucial for the regulation of ferroptosis and warrant further study.

The m^6^A modification has been widely studied in neurological diseases in recent years, and its key role in PCD has gradually come to light, making it the focus of this review. We have also summarized and compared research methods related to m^6^A (**Additional Table 5**). This review highlights the key functions of m^6^A in ferroptosis, cuproptosis, and pyroptosis associated with neurodegenerative diseases. We provide a new perspective on how m^6^A affects the progression of neurodegenerative diseases by participating in PCD and neural regeneration, suggesting that m^6^A will become an important therapeutic target.

**Additional Table 5:** Comparison and discussion of key research methods for m^6^A analysis

Method	Resolution	Advantage	Disadvantage	Reference
M6A-seq	About 100 nt	Pioneering technology that first revealed the whole- transcriptome distribution and potential functions of m^6^A; demonstrated distribution patterns of m^6^A with high specificity	Limited resolution; dependent on annotation of known transcripts; unable to distinguish modification levels of individual transcripts; relying on antibody immunoprecipitation	Dominissini et al., 2012
MiCLIP	Single nucleotide	Accurately identify the specific locations of m^6^A and m^6^Am; independent of predefined sequence motifs, discover m^6^A modifications in atypical DRACH motifs; suitable forvarious RNA modifications	Dependent on specific antibodies with varying effects; incomplete coverage of mutation signals; complex experimental steps and more time-consuming data generation and analysis	Linder et al., 2015
SELECT	Single base	Optimized reaction conditions; distinguish m6A-modified from unmodified adenosine; quantitatively detection the ratio of m6A; no radioactive labeling required; simple operation and high sensitivity; suitable for multiple RNA types	Dependent on known sequence and primer design; enzyme activity may affect the sensitivity and accuracy of detection; require FTO-assisted verification	Xiao et al., 2018
DART-seq	Single nucleotide	Avoid limitations of antibodies; high sensitivity; distinguish m6A from m6A modifications; low cost and rapid; especially suitable for samples with limited RNA	Dependent on cytosines adjacent to m^6^A; cannot directly quantify m^6^A modification levels; background editing exists, requiring strict controls	Meyer, 2019
NOseq	Single base	Use chemical methods to reduce antibody dependence; Applicable to a variety of RNA types; suitable for targeted analysis of specific amplicons	It is impossible to detect m^6^A in the whole transcriptome; xanthosine will significantly inhibit reverse transcription; strong acidic deamination conditions may cause RNA fragmentation	Werner et al., 2021
eTAM-seq	Single base	High conversion efficiency and specificity; high resolution and quantitative ability; suitable for trace samples; retain RNA integrity	Limited detection capacity for hypomethylated sites; require high sequencing depth; complex data analysis	Xiao et al., 2023
Scm6A	Prediction of m^6^A levels based on transcriptome windows	Lower cost and higher efficiency; high accuracy; suitable for various disease and cell type research	Model training relies on known m^6^A regulatory factors and conserved sequence motifs; cannot distinguish dynamic changes in m^6^A; computational models may have potential biases	Li et al., 2024d

DART-seq: Deamination adjacent to RNA modification targets; eTAM-seq: evolved TadA-assisted N^6^-methyladenosine sequencing; m^6^A: N^6^-methyladenosine; MiCLIP: m^6^A individual- nucleotide resolution crosslinking and immunoprecipitation; NOseq: deamination treatment and deamination sequencing; Scm6A: single-cell m^6^A analysis; SELECT: single-base elongation and ligation based PCR amplification method.

In recent years, the development of various technologies, such as omics technology, has led to considerable breakthroughs in the study of the mechanisms underlying neural regeneration. Importantly, many studies have focused on growth factors, microenvironmental barriers, epigenetics, and other aspects, revealing specific mechanisms of neural regeneration in detail. ATP depletion, calcium influx, and ROS generation following nerve injury promote PCD in nerve cells, making PCD a research hotspot. However, most current reviews are limited to a single type of PCD, and the interactions between different forms of cell death and their effects on neural regeneration have not been summarized in detail. This review focuses on the connections between various forms of cell death and neural regeneration, with an emphasis on ferroptosis, cuproptosis, and pyroptosis. Cell death can both promote and inhibit nerve regeneration. On one hand, cell death signals not only clear damaged cells through apoptosis but also promote nerve regeneration through non-apoptotic caspase activity (Klemm et al., 2025). On the other hand, continuous activation of cell death and inflammatory responses inhibits nerve regeneration. Moderate PCD and effective clearance mechanisms can promote nerve regeneration; however, excessive cell death or clearance failure can lead to persistent inflammation and excessive fibrosis, inhibiting axon regeneration and resulting in regeneration failure (Warner et al., 2023). Nerve regeneration also influences cell death. Surviving neurons can affect the process of cell death by secreting related factors (Xu et al., 2024). Importantly, cell death is a core factor that hinders nerve regeneration, and inhibiting neuronal death can significantly promote nerve regeneration and functional recovery. The two processes form a dynamic balance of “death-regeneration” through mechanisms such as the balance of apoptotic molecules, trophic factor secretion, and inflammatory microenvironment regulation (Xu et al., 2024). Regulating this balance is an important target for our research and treatment. This review details the relationships among ferroptosis, cuproptosis, and pyroptosis, as well as the effects of PCD on neural regeneration, and explores the “death-regeneration balance” between them. Although many studies have examined the regulation of epigenetic modifications on neural regeneration, few have focused on the regulation of m^6^A on various PCDs and the death-regeneration balance. We analyzed the important role of m^6^A and innovatively proposed the regulatory axis of m^6^A-PCD-neural regeneration. Our study fills a gap in previous reviews by highlighting the interactions of multiple PCDs and epigenetic modifications, providing a theoretical basis and therapeutic targets for the molecular regulatory network of neural regeneration and the pathogenesis of nervous system diseases. Some experts have proposed novel therapeutic strategies for AD by targeting the conversion of neurodegenerative processes into neural regeneration. These strategies mainly focus on the application of neurotrophic factors and the pharmacological enhancement of endogenous neural regeneration along with synaptic plasticity, aiming to develop more effective small molecule drugs targeting neurotrophic factors in the future (Sun and Alkon, 2024). Other experts have highlighted the critical influence of nervous system maturation on regenerative capacity, revealing significant variations in cellular responses to axonal injury, even among mature neurons sharing similar anatomical and functional characteristics. Scholars in this field emphasize that understanding the developmental programs that drive neural growth and how these programs are regulated to promote synapse formation and neural circuit connections will be an important research direction in neuroscience in the coming years. They also highlight the increasing importance of proteomics technology in advancing these studies. We propose that future research should focus on the specific mechanisms by which m^6^A modification regulates the balance between cell death and regeneration, and we look forward to promoting the development of this field by further clarifying these mechanisms.

This review summarizes a wealth of cutting-edge literature, explores important forms of PCD and their interactions in neurodegenerative diseases, analyzes the dynamic equilibrium between PCD and neural regeneration, and proposes the concept of the death-regeneration balance. Additionally, this review focuses on m^6^A, a key regulatory and therapeutic target, elaborating on the m^6^A-PCD-neural regeneration regulatory axis and its mechanisms in neurodegenerative diseases. Overall, our review provides new insights into the pathogenesis of neurodegenerative diseases and offers a scientific basis for the development of drugs targeting m^6^A modifications and related pathways.

Over the years, many studies have identified various drugs that regulate m^6^A modification as promising therapeutic targets, demonstrating their ability to inhibit PCD, such as ferroptosis, in various disease models. A key factor in their efficacy is the ability of these drugs to cross the blood–brain barrier. Some drugs exhibit good blood–brain barrier permeability and thus hold considerable developmental value. Currently, most m^6^A regulatory drugs are studied in non-neurological disease models, and their mechanisms and effects in neurodegenerative diseases have yet to be verified. The limited application of some effective drugs due to their inability to enter the brain has prompted more researchers to focus on developing advanced drug delivery systems to overcome the blood-brain barrier and improve drug delivery efficiency and targeting. In the future, it will be crucial to strengthen research on the mechanisms of action of m^6^A modification and to explore the effectiveness and safety of m^6^A regulatory drugs for the treatment of neurodegenerative diseases.

Most reviews on neurodegenerative diseases have focused solely on a single form of cell death or have included multiple modes of cell death without addressing epigenetic modifications. There are even fewer reviews on the interactions between various PCDs and their effects on neural regeneration. Therefore, this article innovatively proposes the death-regeneration balance and the m^6^A-PCD-neural regeneration regulatory axis. This study integrates various PCD pathways and their relationship with neural regeneration, highlighting the latest advances in epigenetic modification and drug intervention to elucidate the underlying mechanisms of disease occurrence and development. Additionally, this study identifies potential therapeutic targets and candidate drugs, thereby opening up new avenues for the study of neurodegenerative diseases and addressing existing knowledge gaps.

The m^6^A-PCD-neural regeneration regulatory axis proposed in this review has broad applicability. Its specific mechanism not only explains the pathological mechanisms of neurodegenerative diseases and cognitive impairment but also provides a reference for other central nervous system injuries. The core concept of this regulatory axis is applicable to other diseases such as tumors and inflammatory conditions, offering new insights and a reference for understanding their mechanisms and developing treatments.

## Additional files:

***Additional Table 1:*** *Roles of RNA m*^*6*^*A-regulated ferroptosis in neurodegenerative diseases.*

***Additional Table 2:*** *Role of m*^*6*^*A-regulated pyroptosis in neurodegenerative diseases.*

***Additional Table 3:*** *Therapeutic potential of targeting ferroptosis, cuproptosis, and pyroptosis in neurodegenerative diseases.*

***Additional Table 4:*** *Clinical research on m*^*6*^*A modification and programmed cell death as targets in neurodegenerative diseases.*

***Additional Table 5:*** *Comparison and discussion of key research methods for m*^*6*^*A analysis.*
